# Cell intrinsic heme synthesis is required for the proper activation of naive CD4^+^ T cells

**DOI:** 10.1016/j.isci.2026.118114

**Published:** 2026-11-04

**Authors:** Kazuki Sakurai, Hiroki Kato, Yan Yan, Sayaka Sano, Ryota Saito, Daigo Michimata, Kazuki Hashimoto, Naoya Morota, Yuya Tanaka, Hiroka Komatsu, Tatsuya Watanabe, Daichi Kiba, Hitomi Kashiwagi, Koichi Onodera, Shunsuke Hatta, Yasushi Onishi, Tohru Fujiwara, Takaaki Abe, Fan-Yan Wei, Noriko Fukuhara, Hideo Harigae

**Affiliations:** 1 Department of Hematology, Tohoku University Graduate School of Medicine, Sendai, Japan; 2 Department of Clinical Biology and Hormonal Regulation, Tohoku University Graduate School of Medicine, Sendai, Japan; 3 Department of Laboratory Medicine, Iwate Medical University School of Medicine, Shiwa-gun, Iwate, Japan; 4 Department of Modomics Biology and Medicine, Institute of Development, Aging and Cancer, Tohoku University, Sendai, Japan

**Keywords:** naive CD4^+^ T cells, heme synthesis, glycolysis

## Abstract

Understanding the biology of naive CD4^+^ T cell activation is crucial for comprehending acquired immunity and related disorders. It was identified that the uptake of iron, one of the major micronutrients in mammals, is enormously enhanced during their activation. However, little is known about how iron is utilized in this activation process. Here, we found that the synthesis of heme, one of the major iron-containing metabolites, is indispensable for their activation. Mechanistically, heme supports the interleukin 2-signal transducer and activator of transcription 5 (IL-2-STAT5) signaling to enhance metabolism required for their activation, including glycolysis, which might be achieved by inactivating the repressive transcription factor BACH2. Moreover, several murine autoimmune disorders, derived from CD4^+^ T cells, were ameliorated by heme synthesis inhibition. These findings will pave the way for understanding how T cell activation is controlled by micronutrients through “mitochondria-glycolysis crosstalk”, providing an opportunity to target heme metabolism for the treatment of autoimmune disorders.

## Highlights

• Intrinsic heme synthesis supports naive CD4^+^ T cell activation
• Impaired heme synthesis disturbs glycolytic reprogramming during activation
• CD4^+^ T cell specific heme synthesis impairment attenuates GVHD

## Graphical abstract

**Figure:**
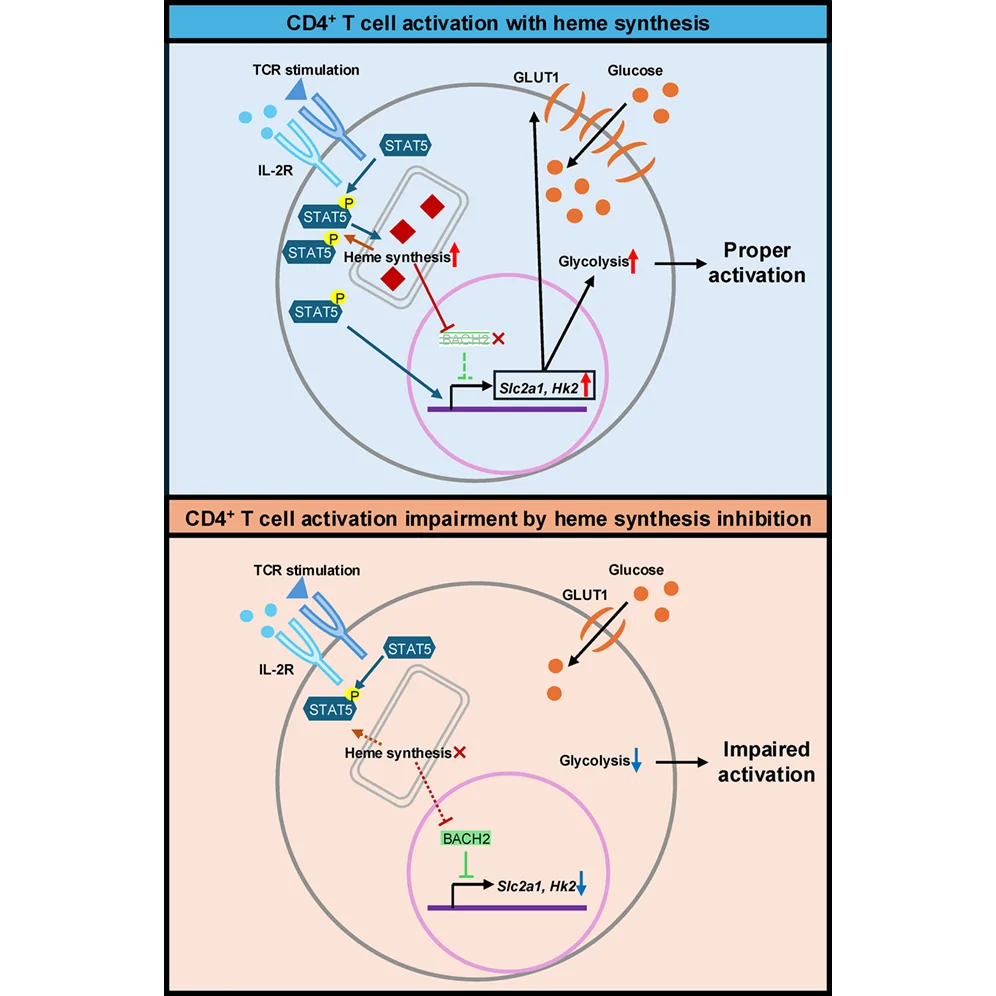

## Teaser

Human metabolism; Immunology; Cell biology

## Introduction

Understanding the biology of T cell hematopoiesis is central to revealing the mechanism by which acquired immunity is properly established. Helper T cells, such as Th1, Th2, and Th17 cells, possess indispensable roles in the orchestration of acquired immunity.^1^^,^^2^ Helper T cells are derived from naive CD4^+^ T cells by T cell receptor (TCR) signaling, co-stimulation factors, and proper cytokine stimulations, such as interleukin 2 (IL-2).^3^^,^^4^^,^^5^ Naive CD4^+^ T cells are mainly dependent on oxidative phosphorylation (OXPHOS) for their energy demand in their dormant state.^6^^,^^7^ On the other hand, glycolysis is enormously enhanced during the naive CD4^+^ T cell activation to meet the rapid increase in their energy demand.^6^^,^^8^ Also, intermediates from glycolysis are utilized to biosynthesize some amino acids (i.e., serine and glycine), as well as nucleotides.^9^ Amino acid metabolism, including serine and glutamine metabolism, and fatty acid oxidation play important roles in the activation and memory formation of naive CD4^+^ T cells.^10^^,^^11^ These metabolic changes are mainly regulated by several activation signaling such as mammalian target of rapamycin complex 1 (mTORC1) and signal transducer and activator of transcription 5 (STAT5) signaling.^12^^,^^13^ However, still little is known about the roles of other nutritional entities to meet the metabolic demand for the activation of naive CD4^+^ T cells.

Iron is an essential micronutrient in cellular biology.^14^^,^^15^ In line with this notion, the expression of iron transporter (transferrin receptor 1: TFRC) is enormously induced during the activation of naive CD4^+^ T cells.^16^ A missense mutation in *TFRC* is reported to cause combined immunodeficiency in humans.^17^ In addition, when cultured with an iron chelator, T cells decreased their OXPHOS and glycolysis.^18^ These studies suggested iron uptake might be indispensable for the proper naive CD4^+^ T cell activation. On the other hand, intracellular iron, especially the free labile iron pool, decreased under TCR stimulation,^19^ suggesting that the intracellular iron might be used to produce iron-containing products during the naive CD4^+^ T cell activation. However, the precise mechanism by which iron supports naive CD4^+^ T cell activation has not fully been elucidated.

Heme is one of the major energetic metabolites synthesized from iron and works as an essential cofactor in the electron transfer chain (respiratory cytochromes).^20^ Heme is also known as a regulator of gene expression by altering the function of transcription factors, including BACH1 and BACH2,^21^ and/or directly binding some genomic loci.^22^ The significance of heme synthesis in hematopoiesis was extensively investigated in the context of erythropoiesis.^23^ However, still little is known about the importance of heme synthesis in naive CD4^+^ T cell activation. Jurkat T cells became more proliferative with the addition of hemin in the culture medium.^24^ In addition, human peripheral T cell proliferation was suppressed by succinyl acetone (SA), which inhibits a delta-aminolevulinic acid dehydratase, one of the heme synthases, *in vitro*.^16^ Although these findings suggest that heme synthesis might be necessary for the proper activation of T cells, the mechanism by which heme synthesis supports the activation of naive CD4^+^ T cells *in vivo* is still obscure.

## Results

### Heme synthase *Cpox* deletion induced CD4^+^ T cell reduction in the spleen

Heme is synthesized via eight catalytic steps with eight individual enzymes (Figure S1A).^25^ To reveal the significance of heme synthesis during the activation of naive CD4^+^ T cells, we revisited the previous data investigating the gene expression alterations in naive CD4^+^ T cells by their activation. Most of the heme synthase expression was increased in naive CD4^+^ T cells by their activation, suggesting that heme synthesis was induced in naive CD4^+^ T cells by their activation (Figure S1B).^26^ These data urged us to investigate the roles of cell-intrinsic heme synthesis during T cell development *in vivo* further. In the heme synthetic pathway, ALAS (ALAS1 and ALAS2) works as a first enzyme at the first part of mitochondrial heme synthesis and coproporphyrinogen oxidase (CPOX) works as a first enzyme in mitochondrial porphyrin processing. Since potential redundancy of ALAS in non-erythroid cells was reported,^27^ we deleted *Cpox* to reveal the role of heme synthesis in CD4^+^ T cells. To this end, we took advantage of the T cell-specific heme synthase-deleted mouse model (*Cpox*^f/f^: CD4-Cre) in which *Cpox* was deleted in a T cell-specific manner.^28^ *Cpox* expression was gradually decreased during the thymic T cell development, and *Cpox* expression was effectively depleted after thymic double-positive (DP) T cells in *Cpox*^f/f^: CD4-Cre (*Cpox*-knockout [KO]) mice compared to *Cpox*^f/f^ (control) mice (Figure 1A). There were no changes in thymic T cell numbers between *Cpox*-KO and control mice (Figures 1B and S1C). On the other hand, splenic CD4^+^ T cell numbers decreased from the naive T cell subset in *Cpox*-KO mice compared to those in control mice (Figures 1C and S1D; Table S1). There were no changes in splenic B cell, myeloid cell, and erythroid cell numbers in *Cpox*-KO mice compared to those in control mice (Figure S1E). Therefore, these data indicated that T cell-specific *Cpox* deletion resulted in reduced T cell numbers in the spleen.

**Figure 1:**
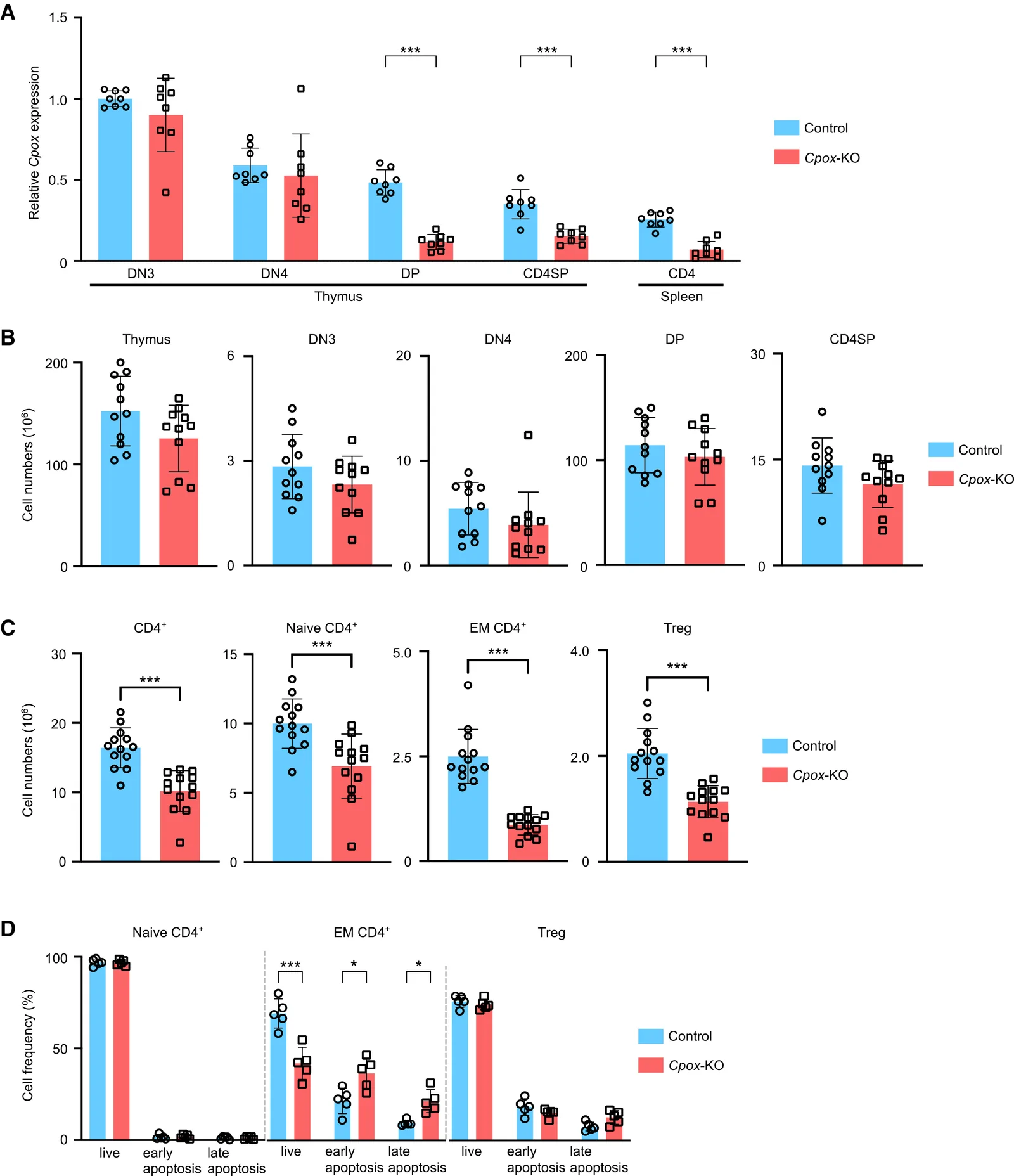
CD4-specific *Cpox* knockout reduced splenic T cells (A) Relative *Cpox* mRNA expression in indicated cells was assessed by qPCR (*n* = 8 mice/group). To obtain the indicated cells, cell sorting from whole thymus or spleen was performed using FACS Aria II as described in STAR Methods. (B) Numbers of the thymus cells and indicated cells of the thymus in control and *Cpox*-KO mice (*n* = 11 mice/group). (C) Numbers of indicated cells of the spleen in control and *Cpox*-KO mice (*n* = 13 mice/group). (D) Apoptosis rates of splenic CD4^+^ T cells *in vivo* were measured by flow cytometry (*n* = 5 mice/group). Each dot represents one mouse/sample. Accumulated data from two or more experiments are shown as mean ± the standard deviation. Statistical analysis was performed using two-tailed Student’s *t* test (∗*p* < 0.05, ∗∗*p* < 0.01, and ∗∗∗*p* < 0.001). Abbreviations: DN, double-negative; DP, double-positive; EM, effector memory, SP, single positive; Treg, regulatory T cell.

To reveal the mechanism by which the splenic CD4^+^ T cell numbers were decreased in *Cpox*-KO mice compared to control mice, we analyzed the apoptotic status of splenic T cells in *Cpox*-KO mice and control mice. This analysis showed that the apoptosis rate was increased in effector memory CD4^+^ T cells but not in naive CD4^+^ T cells (Figures 1D and S1F), suggesting that heme synthesis plays a crucial role in the survival of naive CD4^+^ T cells during their activation.

### Heme synthesis is indispensable for the proper proliferation of naive CD4^+^ T cells *in vitro*

To further reveal the roles of CPOX in naive CD4^+^ T cell activation, we analyzed the expression alteration of *Cpox* during naive CD4^+^ T cell activation. As described earlier, *Cpox* expression was induced in the early phase of naive CD4^+^ T cell activation (Figure 2A), indicating that the heme synthesis was increased during naive CD4^+^ T cell activation. In line with this notion, mass spectrometry revealed that the heme abundance increase from day 0 to day 2 during their activation (Figure 2B). Compared to control, *Cpox*-KO CD4^+^ T cells decreased their heme level (Figure S2A). To further reveal the roles of cell-intrinsic heme synthesis in naive CD4^+^ T cell activation, we analyzed the proliferation ratio of naive CD4^+^ T cells from *Cpox*-KO mice and control mice. The proliferation ratio of *Cpox*-KO naive CD4^+^ T cells was remarkably decreased compared to control cells (Figure 2C). To be noted, this suppression was rescued by hemin addition (Figure 2D), but not by sodium ferrous citrate (SFC) addition (Figure S2B), indicating that iron utilization for heme synthesis is required for the proper activation of naive CD4^+^ T cells. SA treatment also suppressed the proliferation ratio of naive CD4^+^ T cells in a concentration-dependent manner (Figure S2C), and this suppression was, at least partially, rescued by hemin addition (Figure S2D). Collectively, these data indicated that heme synthesis from iron is indispensable for the proper activation of naive CD4^+^ T cells.

**Figure 2:**
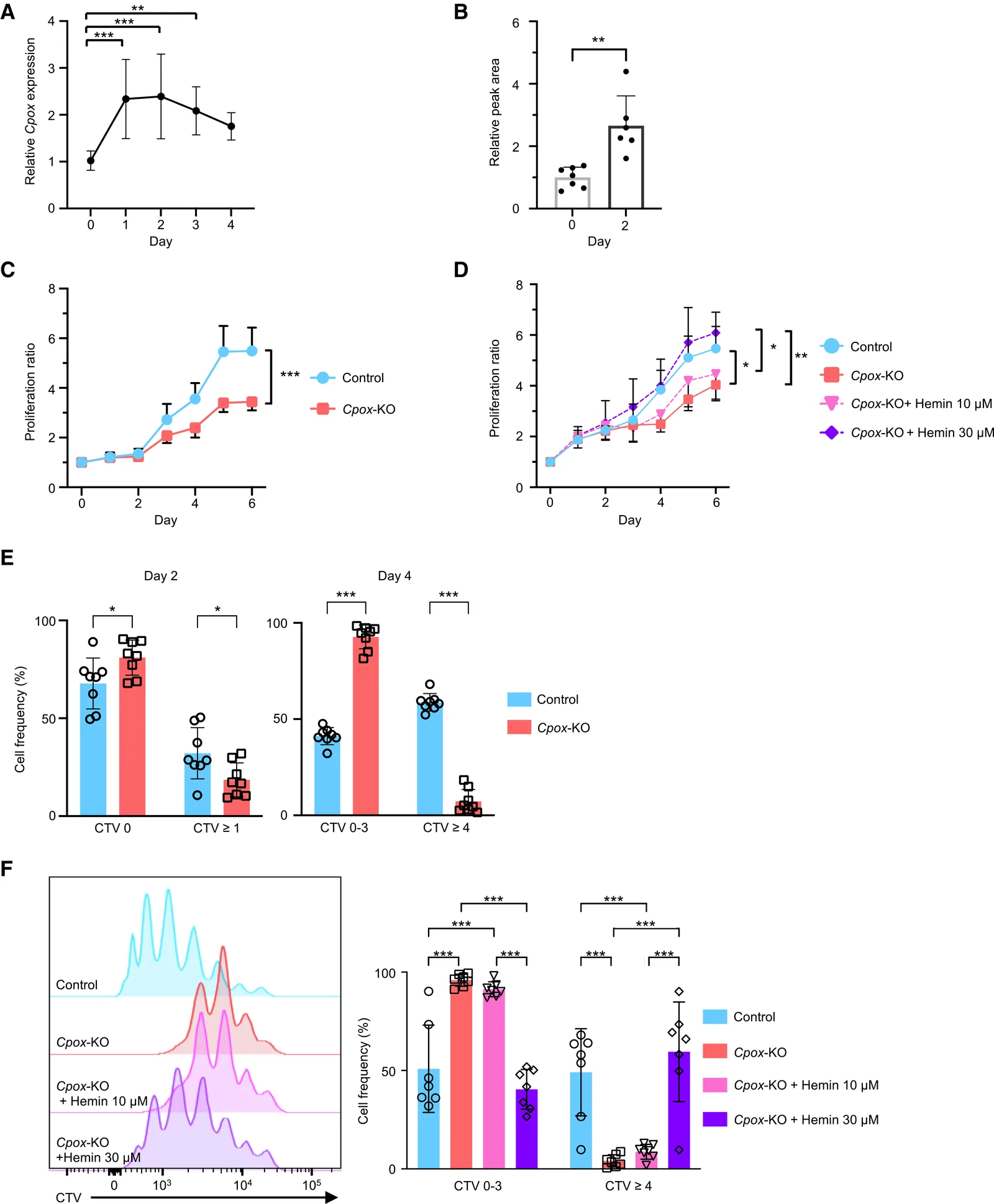
Inhibition of cell intrinsic heme synthesis suppressed naive CD4^+^ proliferation *in vitro* (A) Relative *Cpox* mRNA expression during wild-type naive CD4^+^ T cell activation was analyzed by qPCR (*n* = 6 mice). We collected cultured bulk CD4^+^ T cells and then extracted mRNA as described in STAR Methods. (B) Intracellular heme concentration of wild-type naive CD4^+^ T cells and *in vitro* activated CD4^+^ T cells on day 2 was assessed by mass spectrometry (*n* = 6 mice on day 0 and *n* = 5 mice on day 2). To obtain the indicated cells, naive CD4^+^ T cells were collected by magnetic cell sorting on day 0, then we sorted CD4^+^ T cells by flow cytometry just after magnetic cell sorting or 2 days after activation (on day 2). (C) Proliferation ratio of naive CD4^+^ T cells during their *in vitro* activation (*n* = 10 mice/group). (D) Proliferation ratio of naive CD4^+^ T cells with or without hemin addition (*n* = 7 mice/group). (E) The frequencies of cell trace staining on day 2 and 4 of *in vitro* activated CD4^+^ T cells (*n* = 8 mice/group). (F) Representative cell trace staining histogram (left) and the frequencies (right) of cell trace staining on day 4 of activated CD4^+^ T cells with or without hemin addition (*n* = 7 mice/group). The *y* axis of the histogram showed the percentage of the maximum signal (% of max). Each dot represents one mouse/sample. Accumulated data from two or more experiments are shown as mean ± the standard deviation unless otherwise mentioned. Statistical analysis was performed using two-tailed Student’s *t* test (∗*p* < 0.05, ∗∗*p* < 0.01, and ∗∗∗*p* < 0.001) to compare two groups (B). Comparison of three or more groups was performed by one-way ANOVA test followed by Dunnett’s (A) or Tukey (F) multiple-comparison tests (∗*p* < 0.05, ∗∗*p* < 0.01, and ∗∗∗*p* < 0.001) and two-way repeated measures ANOVA test followed by Sidak (C) or Tukey (D) multiple-comparison tests for proliferation ratio whose primary endpoint was on day 6 (∗*p* < 0.05, ∗∗*p* < 0.01, and ∗∗∗*p* < 0.001).

To reveal the mechanism by which heme synthesis supports the proper activation of naive CD4^+^ T cells, we performed cell trace staining to analyze the cell division status. This analysis demonstrated a decrease in the number of cell divisions in *Cpox*-KO naive CD4^+^ T cells compared to the control cells on day 2 and day 4 (Figures 2E and S2E). The addition of hemin to the culture medium could completely rescue this phenomenon at a concentration of 30 μM (Figure 2F). Also, the early apoptosis rate in control cells was slightly lower than that in *Cpox*-KO cells, and that was not different from *Cpox*-KO cells with hemin (Figure S2F). We also performed the cell cycle analysis using bromodeoxyuridine (BrdU) and 7-amino-actinomycin (7-AAD) staining. *Cpox*-KO reduced the percentage of G0/G1 phase, which was rescued by hemin (Figure S2G). Further analysis is needed to confirm if this small cell cycle progression alteration in *Cpox*-KO cells actually causes their apoptosis, but the increased apoptosis in *Cpox*-KO cells might be triggered by cell cycle progression alteration in *Cpox*-KO cells. Collectively, these data indicated that heme synthesis is indispensable for the proper cell proliferation in naive CD4^+^ T cells *in vitro*.

### Cell-intrinsic heme synthesis is crucial for the proper cell activation of naive CD4^+^ T cells *in vivo*

Next, we sought to reveal whether cell-intrinsic heme synthesis is required for the proper cell activation of naive CD4^+^ T cells *in vivo*. To this end, we conducted adoptive T cell transfer experiments. It has been reported that acute lymphopenia, such as observed in an irradiated host, induced moderate proliferation of transferred CD4^+^ T cells. On the other hand, chronic lymphopenia, such as observed in a Rag-deficient host, induced aggressive proliferation of transferred CD4^+^ T cells.^29^^,^^30^ First, we transplanted *Cpox*-KO or control naive CD4^+^ T cells into irradiated (5.0 Gy) CD45.1^+^ mice. After 7 days of transplantation, splenic T cells were analyzed (Figure 3A). The proportion of CD45.2^+^ cells in the CD4^+^ T cell subset was decreased in *Cpox*-KO cell transplanted mice compared to control cell transplanted mice (Figure 3B). When transferred cell trace-stained *Cpox*-KO naive CD4^+^ T cells, they declined rapid proliferation compared to control cells (Figures 3C and S3A), indicating that the rapid proliferation of transferred naive CD4^+^ T cells were impaired by *Cpox* deletion. We also performed cell transplantation analysis using *Rag2*-KO mice (Figure 3D) in which mature lymphoid cells were inherently absent.^31^ The number of CD4^+^ T cells in the spleen was significantly lower in the mice who obtained the *Cpox*-KO cells than those who obtained the control cells and this difference enlarged from day 7 to day 14 (Figures 3E, S3B, and S3C). Upon activation, naive CD4^+^ T cells lost their CD62L expression,^32^ the geometric mean fluorescent intensity (MFI) was significantly lower in control CD4^+^ T cells on day 7 (Figure 3F), while that of *Cpox*-KO cells remained high on day 14 (Figure S3D). Collectively, these data indicated that the cell-intrinsic heme synthesis is crucial for the proper cell activation and proliferation of naive CD4^+^ T cells *in vivo*.

**Figure 3:**
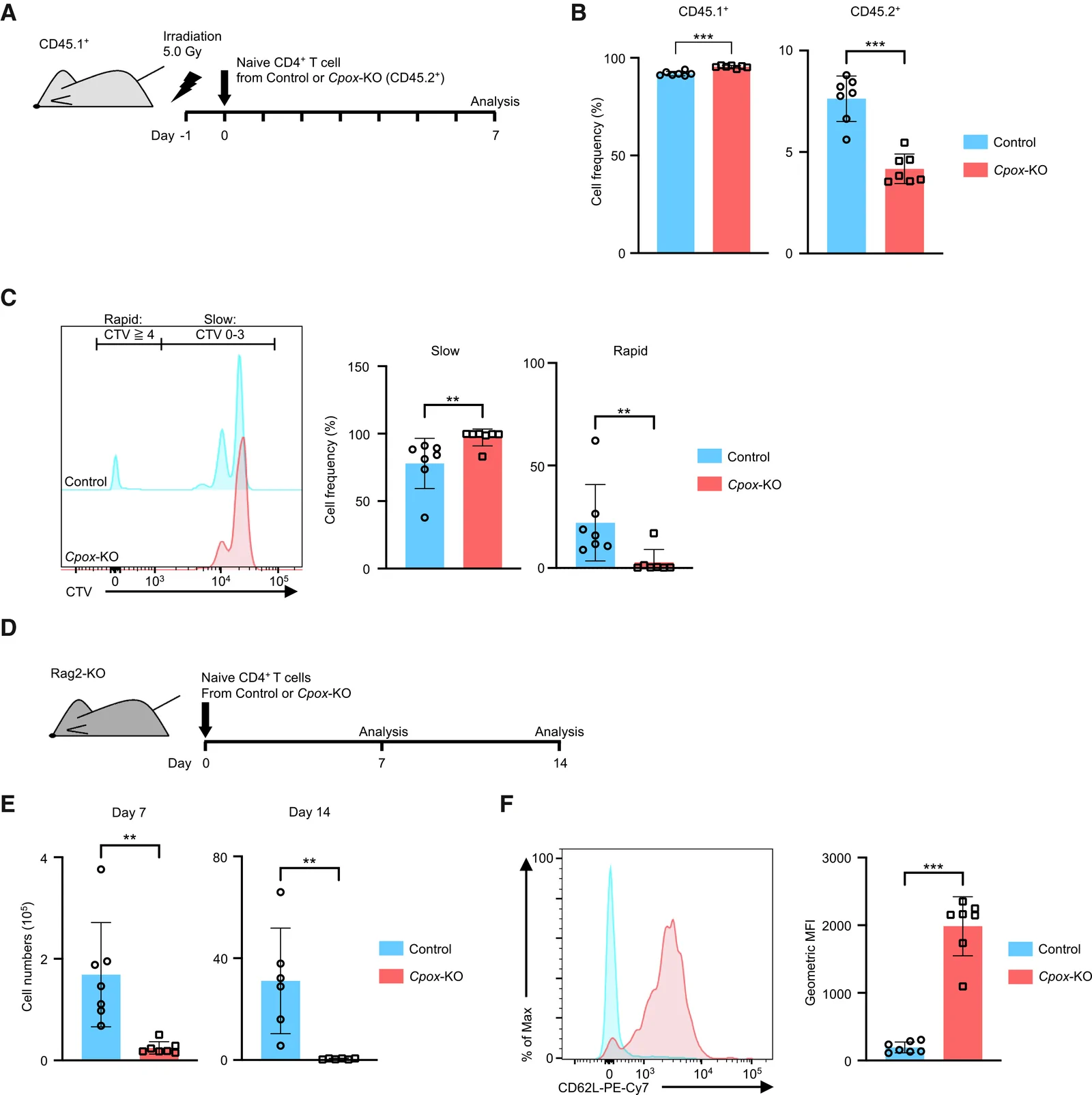
*Cpox*-KO suppressed cell division during homeostatic lymphoproliferation of naive CD4^+^ T cells *in vivo* (A) The schema of control or *Cpox*-KO mice-derived naive CD4^+^ T cells adoptive transfer to irradiated CD45.1^+^ mice. (B) Cell frequency of CD45.1^+^ and CD45.2^+^ cells in the spleen of recipient mice (CD45.1^+^) (*n* = 7 mice/group). (C) Representative histogram of cell trace violet staining by flow cytometry (left) and cell frequency (right) of slow and rapid proliferation, which was defined by cell division 0–3 times and four or more times (*n* = 7 recipient mice/group). The *y* axis of the histogram showed the percentage of the maximum signal (% of max). (D) The schema of control or *Cpox*-KO mice-derived naive CD4^+^ T cells adoptive transfer to *Rag2*-KO mice. (E) Cell numbers of CD4^+^ T cells of the spleen in transferred *Rag*2-KO mice on day 7 (*n* = 7 recipient mice/group) and day 14 (*n* = 6 recipient mice/group). (F) Representative histogram of CD62L expression by flow cytometry (left) and geometric mean fluorescent intensity of CD62L (right) of CD4^+^ T cells in transferred *Rag2*-KO mice on day 7 (*n* = 7 recipient mice/group). Each dot represents one mouse/sample. Accumulated data from two or more experiments are shown as mean ± the standard deviation. Statistical analysis was performed using two-tailed Student’s *t* test (∗*p* < 0.05, ∗∗*p* < 0.01, and ∗∗∗*p* < 0.001).

### Heme synthesis is required for the proper induction of glycolysis-related gene expression during naive CD4^+^ T cell activation

We sought to reveal the mechanism by which proper activation was impaired in *Cpox*-KO naive CD4^+^ T cells under their activation condition. To this end, we performed a comprehensive transcriptomic analysis of *Cpox*-KO and control naive CD4^+^ T cells with or without activation (day 0, day 2, and day 4). As expected, the expression of many genes was changed during naive CD4^+^ T cell activation (control day 0 vs. control day 2: 6,995 genes, control day 0 vs. control day 4: 5,824 genes (|log_2_FC| > 0.585, false discovery rate [FDR] <0.1). The expression of some heme synthase genes, including *Cpox*, was increased by the activation in control cells (Figure 4A). Gene set enrichment analysis (GSEA)^33^^,^^34^ revealed that hypoxia- and glycolysis-related gene sets were upregulated by the activation as previously described (Figures 4B and S4A).^35^ Gene expression changes were limited between *Cpox*-KO and control naive CD4^+^ T cells before the activation (control day 0 vs. *Cpox*-KO day 0: 29 genes, including *Cpox* (|log_2_FC| > 0.585, FDR < 0.1). In contrast, gene expression changes became apparent after the activation between *Cpox*-KO and control naive CD4^+^ T cells (control day 2 vs. *Cpox*-KO day 2: 1,245 genes, control day 4 vs. *Cpox*-KO day 4: 997 genes (|log_2_FC| > 0.585, FDR < 0.1) (Figure S4B). GSEA revealed that hypoxia- and glycolysis-related gene sets were downregulated in *Cpox*-KO cells (Figures 4C and S4C). Database for Annotation, Visualizarion and Discovery (DAVID) analysis also revealed that the hypoxia-induced pathway and glycolysis pathway were downregulated in *Cpox*-KO cells (Figure S4D).^36^ Upon extracting glycolysis-related genes from these data, the glycolysis pathway was upregulated by the activation in control cells and downregulated in *Cpox*-KO cells (Figure 4D). We also validated the expression of glucose transporter 1 (*Slc2a1*) and the key glycolytic enzyme hexokinase II (*Hk2*) by RT-qPCR. We revealed that *Cpox*-KO reduced the mRNA expression of both genes, which was rescued by hemin addition (Figure 4E). Collectively, these data revealed that heme synthesis is required for the proper induction of glycolysis-related gene expression during naive CD4^+^ T cell activation.

**Figure 4:**
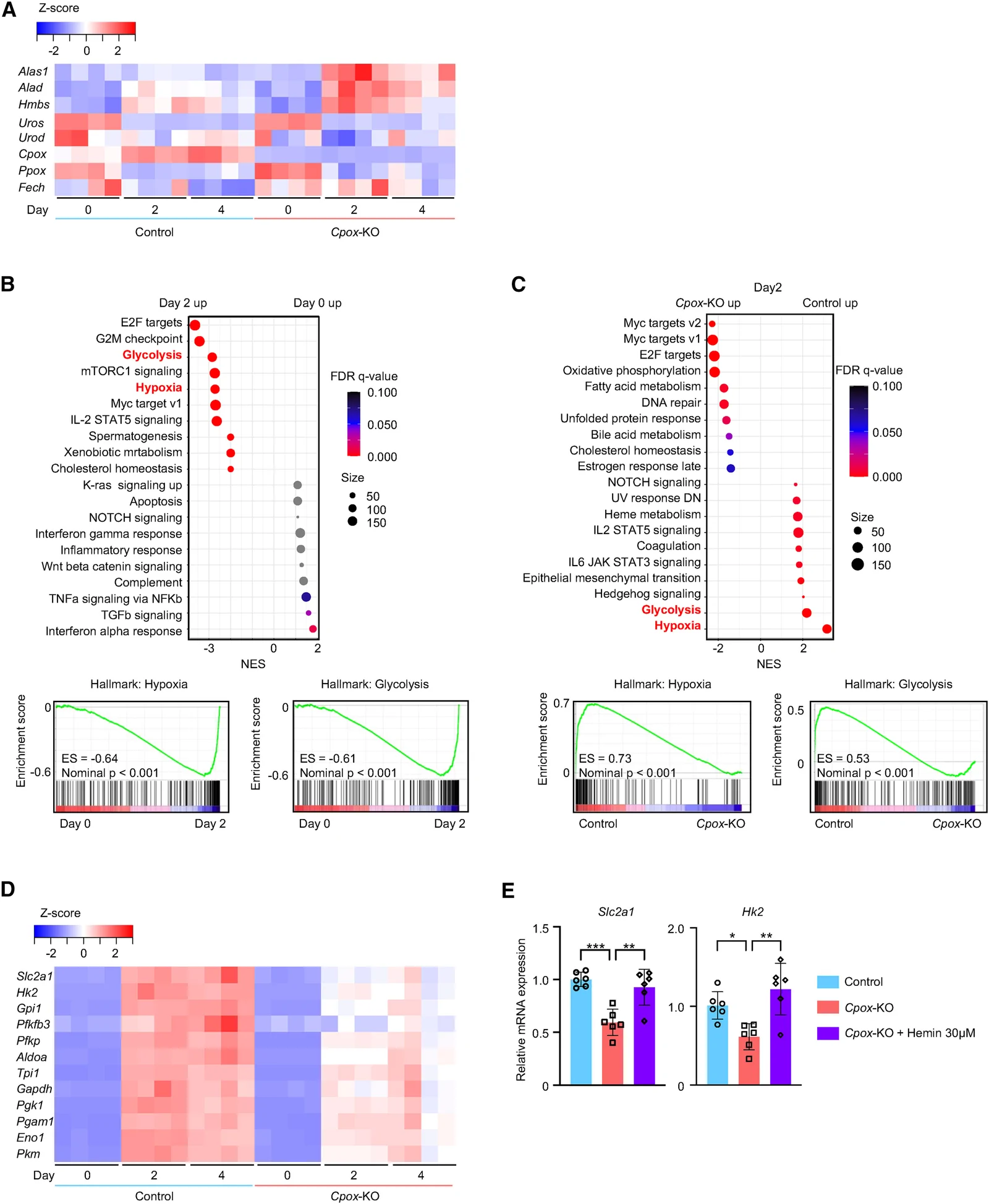
Cell intrinsic heme synthesis plays an important role in the glycolysis pathway during naive CD4^+^ T cell activation (A) Heme synthase gene expression of control and *Cpox*-KO naive CD4^+^ T cells during their activation at the indicated time points. (B) Top and bottom 10 gene sets from GSEA mouse hallmark gene sets comparing day 0 and 2 of control naive CD4^+^ T cell activation (top). Enrichment plots of hypoxia and glycolysis (bottom). (C) Top and bottom 10 gene sets from GSEA mouse hallmark gene sets comparing control and *Cpox*-KO activated CD4^+^ T cell on day 2 (top). Enrichment plots of hypoxia and glycolysis (bottom). (D) Glycolysis-related gene expression in control and *Cpox*-KO naive CD4^+^ T cells during their activation at the indicated time points. (E) Relative mRNA expression of *Slc2a1* and *Hk2* on naive CD4^+^ T cells activation (day 2) (*n* = 6 mice/group). We collected cultured bulk CD4^+^ T cells and then extracted mRNA as described in STAR Methods. Each dot represents one mouse/sample. Accumulated data from two or more experiments are shown as mean ± the standard deviation. Statistical analysis was performed using one-way ANOVA test followed by Tukey multiple-comparison test (∗*p* < 0.05, ∗∗*p* < 0.01, and ∗∗∗*p* < 0.001).

### Heme synthesis is required for the proper induction of glycolysis in the activation of naive CD4^+^ T cells

The data described earlier indicate that heme synthesis is required for the proper induction of glycolysis in the activation of naive CD4^+^ T cells. Therefore, we assessed CD4^+^ T cell metabolism by Seahorse XF Pro analyzer. As previously reported and expected from our transcriptomic data described earlier,^6^^,^^8^ the glycolysis measured by extracellular acidification rate (ECAR) was enormously enhanced upon T cell activation and impaired by *Cpox* deletion, which was rescued by hemin (Figure 5A). These data indicated that heme synthesis is required for the proper induction of glycolysis during CD4^+^ T cell activation.

**Figure 5:**
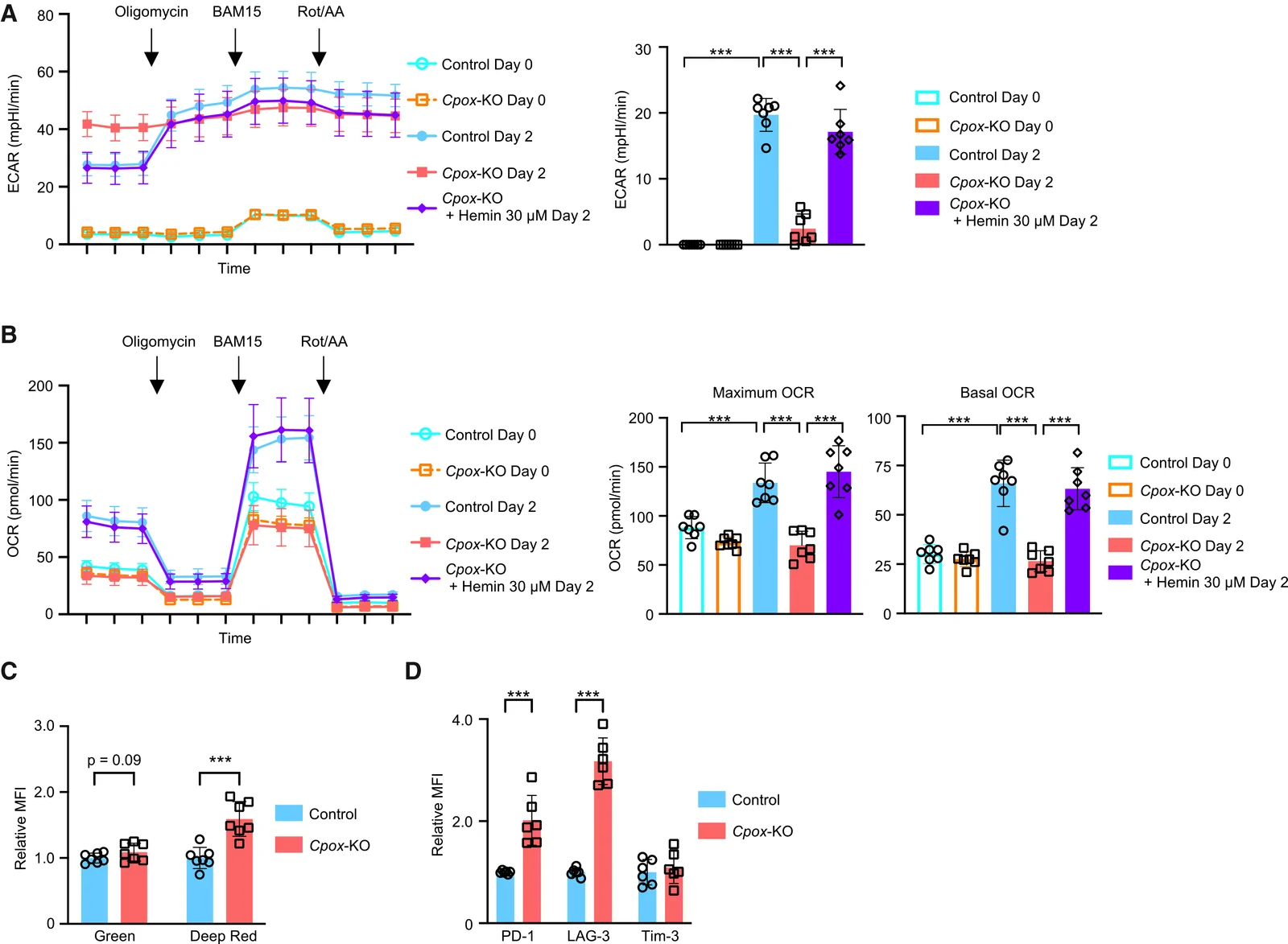
Cell intrinsic heme synthesis promotes glycolysis and links to fitness during naive CD4^+^ T cell activation (A) ECAR assay from naive (day 0, *n* = 7 mice/group) or activated (day 2, *n* = 7 mice/group) CD4^+^ T cells using Seahorse. The left line graph showed the time course, and the right bar graph showed the calculated ECAR. (B) OCR assay from naive (day 0, *n* = 7 mice/group) or activated (day 2, *n* = 7 mice/group) CD4^+^ T cells using Seahorse. The left line graph showed the time course, and the right bar graph showed the calculated basal and maximum OCR. (C) Relative geometric mean fluorescent intensity of mitotracker green and deep red in activated CD4^+^ T cells on day 4 (*n* = 7 mice/group). (D) Relative geometric mean fluorescent intensity of inhibitory receptors (PD-1, LAG-3, and Tim-3) in activated CD4^+^ T cells on day 4 (*n* = 6 mice/group). Each dot represents one mouse/sample. Accumulated data from two or more experiments are shown as mean ± the standard deviation. Statistical analysis was performed using one-way ANOVA test followed by Tukey multiple-comparison test for three or more groups (A, B) and two-tailed Student’s *t* test for (C) and (D) (∗*p* < 0.05, ∗∗*p* < 0.01, and ∗∗∗*p* < 0.001).

We also analyzed the OXPHOS by measuring the basal and maximum oxygen consumption rate (OCR). OXPHOS measured by OCR was also enhanced by T cell activation and impaired by *Cpox* deletion, which was rescued by hemin (Figure 5B). To be noted, the induction rate of glycolysis during the T cell activation was much larger than that of OXPHOS, suggesting that the glycolysis possess more important roles in the T cell activation as previously described.^37^ Intriguingly, the abundance of complex III protein expression, which included heme as one of its components, changed similarly to OXPHOS activity but not its related transcripts (Figures 4C, S4C, S5A, and S5B), suggesting that a feedback system might exist to support the impaired OXPHOS activity in *Cpox*-KO cells at the transcriptional level. We also checked mitotracker staining of green and deep red, which reflected mitochondrial mass and membrane potential, respectively.^38^ *Cpox*-KO had slightly higher geometric MFI of green and higher MFI of deep red (Figures 5C and S5C). These conflicts were also seen in exhausted T cells,^39^ then we confirmed several exhaustion markers. Relative MFI of programmed cell death protein 1 (PD-1) and lymphocyte activation gene 3 (LAG3) was statistically higher in *Cpox*-KO (Figures 5D and S5D). Therefore, heme synthesis is required for the proper induction of glycolysis in the activation of naive CD4^+^ T cells while maintaining their metabolic fitness.

### Heme synthesis possesses important roles in STAT5 signaling during naive CD4^+^ T cell activation

We next asked why heme synthesis is required to drive glycolysis during the activation of naive CD4^+^ T cells. mTORC1 pathways,^12^ STAT5 signaling,^13^ hypoxia-induced factor 1 alpha (Hif1a) signaling,^40^ and c-*myc*^41^ were identified as important regulators of glycolysis. Since GSEA revealed that c-*myc* target genes were enriched in *Cpox*-KO cells (Figure 4C), we focused on other signaling. There were no particular changes in pS6 level and HIF1A expression between *Cpox*-KO and control cells (Figures S6A and S6B). On the other hand, p-STAT5 was higher in control cells than in *Cpox*-KO cells, which was rescued by hemin (Figure 6A). We also re-analyzed p-STAT5/STAT5 ratio, the phosphorylation ratio was significantly lower in *Cpox*-KO and partially rescued by hemin (Figure S6C). These findings indicated that heme might support the activation of STAT5 signaling to enhance glycolysis.

**Figure 6:**
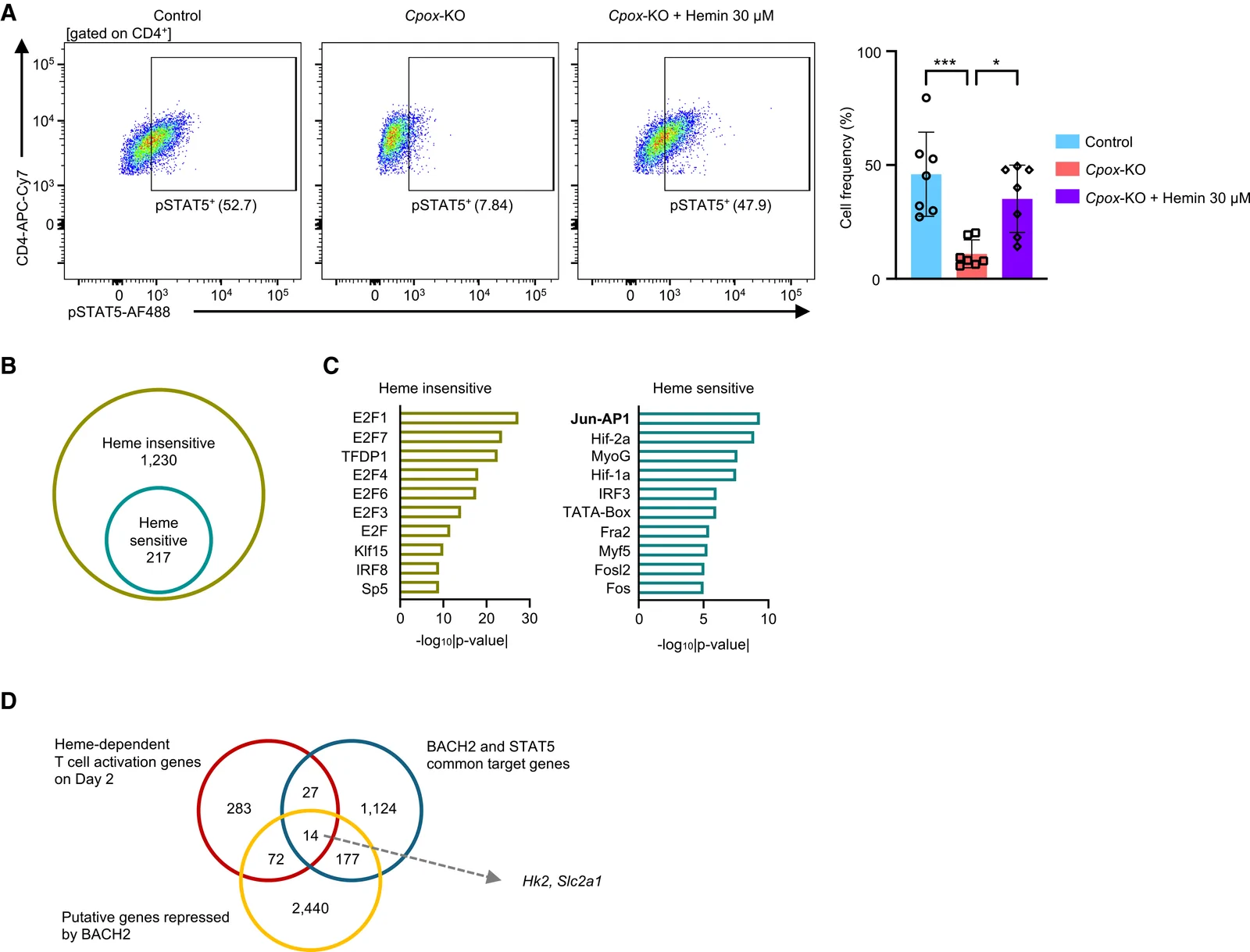
Heme synthesis supports IL-2-STAT5 signaling (A) Representative plots of p-STAT5 stain by flow cytometry (left) and cell frequency (right) of p-STAT5^+^ cells in activated naive CD4^+^ T cells on day 2 (*n* = 7 mice/group). Each dot represents one mouse/sample. Accumulated data from two or more experiments are shown as mean ± the standard deviation Statistical analysis was performed using two-tailed Student’s *t* test (∗*p* < 0.05, ∗∗*p* < 0.01, and ∗∗∗*p* < 0.001). (B) Merged figure of our RNA sequence data and previous data. The olive ring shows genes which met both upregulation (|log_2_FC| > 0.585) in control day 2 compared to control day 0 and enrichment of STAT5 ChIP-seq (GSE207265). Green ring exhibits the genes in olive group which satisfied upregulation (|log_2_FC| > 0.585) in control compared to *Cpox*-KO cells on day 2, named as “heme sensitive”. The genes included in olive group but not green were named as “heme insensitive”. (C) Top 10 motif lists of HOMER analysis of heme-insensitive genes (left) and heme-sensitive genes (right). (D) Merged figure of our RNA sequence data and previous data. The red ring shows merged upregulated genes (|log_2_FC| > 0.585) of control day 2 compared to control day 0, and control cells compared to *Cpox*-KO cells (day 2) using our RNA-seq data. The blue ring shows merged STAT5 ChIP-seq (GSE207265) and three BACH2 ChIP-seq data (GSE45975, GSE126800, and GSE127695). Genes that had common enrichment on the same genomic loci in all four ChIP-seq data were shown. The yellow ring shows upregulated genes (FC > 1.25) in *Bach2*-KO T cell versus wild-type T cell RNA-seq data (GSE123350). Upregulated genes (fold change [FC] > 1.25) in *Bach2*-KO T cells compared to wild-type T cells were shown. Fourteen extracted genes were listed in Table S2A.

To reveal the mechanism by which heme supports the activation of STAT5 signaling during T cell activation, we investigated differentially expressed genes (DEGs) defined as genes with |log2FC| > 0.585 and FDR < 0.1 (day 2 > day 0 in control cells and control cells > *Cpox*-KO cells in day 2) from our transcriptomic data with STAT5 target genes identified by previous chromatin immunoprecipitation sequencing (ChIP-seq) data (GEO Database: GSE207265).^42^ In 1,447 genes extracted as upregulated genes during control naive CD4^+^ T cell activation and STAT5 target, 217 genes (15.0%) seemed to be heme sensitive (Figure 6B). We conducted Hypergeometric Optimization of Motif EnRichment (HOMER) analysis to these genes.^43^ Heme-sensitive genes had Jun-AP1 consensus DNA sequence (TGA [C/G] TCA), which was not enriched in heme-insensitive genes (Figure 6C). Since Jun-AP1 motif was included in Maf recognition elements (MAREs), and BACH2 was reported to bind to this motif in CD4^+^ T cells,^44^^,^^45^ we focused on BACH2, a unique transcription factor that is directly suppressed by heme and whose importance in T cell differentiation has been widely reported.^46^^,^^47^ To this end, we integrated DEGs (day 2 > day 0 in control cells, and control cells > *Cpox*-KO cells in day 2) from our transcriptomic data with BACH2 and STAT5 common target genes identified by previous ChIP-seq data (GEO Database: BACH2; GSE45975,^47^ GSE126800,^48^ and GSE127695^49^; STAT5; GSE207265^42^). We also merged the upregulated genes in *Bach2*-KO naive CD4^+^ T cells (GEO Database: GSE123350^50^). As a result, 14 genes including *Slc2a1* and *Hk2* were identified (Figures 6D and S6D; Table S2A). *Slc2a1* and *Hk2* were also identified using DEGs (day 4 > day 0 in control cells, and control cells > *Cpox*-KO cells in day 4) (Figure S6E; Table S2B). Some of the other genes extracted from each analysis, such as *Tnfrsr8*, *Il2rb*, and *Ahnak*, were also related to CD4^+^ T cell activation and inflammation.^51^^,^^52^^,^^53^^,^^54^ These proteins might mutually accelerate CD4^+^ T cell activation and then reinforce IL-2-STAT5 signaling. In terms of heme synthesis, STAT5 might regulate four out of eight heme synthases, including *Cpox*, and also *Tfrc* (Figure S6F). We validated mRNA expressions of these four heme synthases, which were upregulated on day 2 (Figures 2A and S6G). These ChIP-seq data (GEO Database: GSE207265) reinforced our results of heme abundance increase during naive CD4^+^ T cell activation as described in Figure 2B. These data indicated that heme might support the activation of STAT5 target genes by repressing BACH2 function.

### Heme synthesis inhibition might prevent over-inflammatory reactions *in vivo*

Finally, we sought to reveal the importance of heme synthesis during the activation of naive CD4^+^ T cells *in vivo*. To this end, we took advantage of the adoptive T cell transfer colitis model in *Rag2*-KO mice.^55^ We analyzed body weight changes after adoptive transfer for 8 weeks in *Rag2*-KO mice. Although control cell-transferred *Rag2*-KO mice showed a marked decrease in their body weight after 3 weeks, *Cpox*-KO cell-transferred *Rag2*-KO mice showed no particular reduction in their body weight (Figure 7A), suggesting that the adoptive T cell transfer colitis was limited in *Cpox*-KO cell-transferred *Rag2*-KO mice. In line with this notion, pathological assessment of hematoxylin-eosin staining revealed that lymphocytes infiltrated more in the lamina propria, goblet cells were more decreased, and crypt apoptosis was more obvious in control cell-transferred *Rag2*-KO mice than in *Cpox*-KO cell transferred *Rag2*-KO mice (Figure 7B).

**Figure 7:**
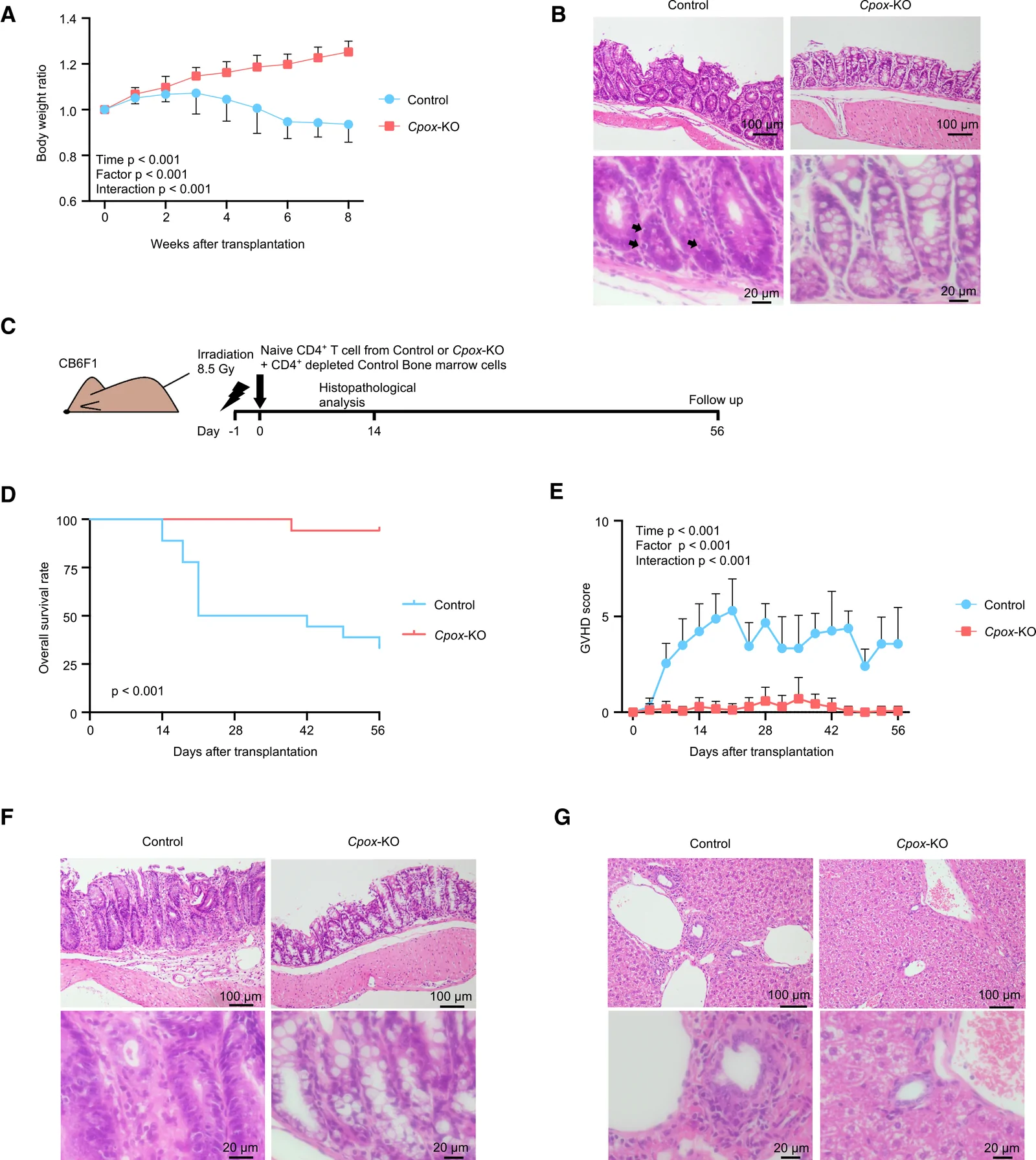
Impairment of cell intrinsic heme synthesis suppressed autoimmune-mediated inflammation and GVHD (A) The change of body weight of *Rag2*-KO mice after naive CD4^+^ T cells adoptive transfer (control; *n* = 10 recipient mice, *Cpox*-KO; *n* = 9 recipient mice, experiments = 3). The comparison was assessed by two-way mixed-effects (group and time) measures ANOVA test followed by Sidak multiple-comparison test. (∗*p* < 0.05, ∗∗*p* < 0.01, and ∗∗∗*p* < 0.001). (B) Representative picture of pathological assessment of colon by Hematoxylin-Eosin staining at 4 weeks after adoptive transfer. Upper images were taken at low magnification (10×) and lower images were taken at high magnification (40×). Arrows on the left side indicated crypt apoptosis. (C) The schema of this GVHD model. (D) Overall survival rate after transplantation (control; *n* = 18 recipient mice, *Cpox*-KO; *n* = 17 recipient mice, experiments = 4). Statistical analysis was performed using log rank test. (E) The change of GVHD score after transplantation (control; *n* = 18 recipient mice, *Cpox*-KO; *n* = 17 recipient mice, experiments = 4). The comparison was assessed by two-way mixed-effects (group and time) measures ANOVA test followed by Sidak multiple-comparison test (∗*p* < 0.05, ∗∗*p* < 0.01, and ∗∗∗*p* < 0.001). (F) Representative picture of pathological assessment of colon by hematoxylin-eosin staining on day 14. Upper images were taken at low magnification (10×) and lower images were taken at high magnification (40×). (G) Representative picture of pathological assessment of liver by hematoxylin-eosin staining on day 14. Upper images were taken at low magnification (10×) and lower images were taken at high magnification (40×). All pathological assessments were performed at least two experiments using four or more samples. Scale bars represent 100 μm in the low magnification images and 20 μm in the high magnification images.

We further analyzed the effect of *Cpox* deletion in naive CD4^+^ T cells using a graft-versus-host-disease (GVHD) model (Figure 7C). Recipient mice, CB6F1, were generated from BALB/c and B6 mates. Recipient mice were lethally irradiated 1 day before transplantation. Donor mice were control or *Cpox*-KO mice, both of which were on a B6 background. Naive CD4^+^ T cells either from control or *Cpox*-KO mice, and CD4^+^ T cell-depleted bone marrow cells from control mice were transplanted to the same sex recipients. The overall survival rate was lower in the control naive CD4^+^ T cell transferred group than the *Cpox*-KO cell transferred group (Figure 7D). GVHD score exacerbated at around day 14 in the control group (Figure 7E). Physical findings showed hunched posture and ruffled fur (Figure S7A) in the control group. We assessed the histopathology of the colon and liver. More severe lymphocyte infiltration in the lamina propria, crypt regeneration, and epithelial cell apoptosis were observed in the control group than in the *Cpox*-KO group, which was similar to the results of adoptive T cell transfer to the *Rag2*-KO mice model (Figure 7F). Lymphocytes also infiltrated into perivascular or periductular lesions more prominently in the control group than the *Cpox*-KO group (Figure 7G). These pathological findings were characteristic of murine GVHD,^56^ and *Cpox*-KO naive CD4^+^ T cells caused less GVHD.

Collectively, these findings indicated that impairments of heme synthesis in naive CD4^+^ T cells might prevent life-threatening inflammatory reactions *in vivo*.

## Discussion

Little was known about the importance of heme synthesis in naive CD4^+^ T cell activation *in vivo*. In this study, we revealed that the cell-intrinsic heme synthesis is required for the proper activation of naive CD4^+^ T cells *in vivo*. T cell proliferation disorders have been observed under conditions of iron starvation or iron chelation.^19^^,^^57^ However, it was not fully clear how iron was utilized to activate T cells. We revealed that heme, as one of the major iron-containing metabolites, plays a crucial role not only in the induction of OXPHOS but also in the induction of glycolysis upon naive CD4^+^ T cell activation. Exogenous heme addition rescued the *Cpox*-KO T cells in a specific concentration *in vitro* (30 μM hemin). On the other hand, *Cpox*-KO T cells require intrinsic heme synthesis *in vivo*. These results suggest that serum-free heme concentration is not enough for *Cpox*-KO T cells to uptake serum-free heme and overcome their intrinsic heme synthesis deficiency *in vivo*. This finding is in line with the notion that serum-free heme is immediately trapped by heme scavenger proteins, such as hemopexin, to keep the serum-free heme concentration low enough to prevent heme-dependent toxicity, including oxidative stress.^58^^,^^59^ Whether T cells can use exogenous heme for their activity *in vivo* when there is enough heme remains to be elucidated. Therefore, further analysis is required to reveal the significance of iron-heme metabolism, especially in the microenvironment, for the proper T cell activation.

Effect of *Cpox* deletion is much greater in effector memory CD4^+^ T cells than naive CD4^+^ T cells, suggesting that heme synthesis may be dispensable for naive CD4^+^ T cell survival per se. For instance, no particular difference in apoptosis rates between control and *Cpox*-KO naive CD4^+^ T cells was observed (Figure 1D). On the other hand, naive CD4^+^ T cell numbers were decreased in *Cpox*-KO mice (Figure 1C). Since naive CD4^+^ T cell subsets consist of heterogeneous cells in which naive CD4^+^ T cells that are exactly free from activation (extremely naive CD4^+^ T cells) and naive CD4^+^ T cells that are under activation (transitioning naive CD4^+^ T cells) should exist, it is possible that only the number of transitioning naive CD4^+^ T cells might be decreased in *Cpox*-KO mice. This concept is also supported by the recent single-cell transcriptomic analysis targeting human resting CD4^+^ T cells, which showed the existence of a T cell effectorness gradient.^60^ Heme synthesis might play a significant role in the metabolic switch from extremely naive CD4^+^ T cells to transitioning naive CD4^+^ T cells. Further analysis, including single-cell transcriptomic analysis, will help us nail down the point at which heme acts as a key driver of T cell activation.

OXPHOS might be reduced in *Cpox*-KO cells due to the direct loss of intracellular heme abundance, because some heme-dependent proteins, such as cytochromes, are required for OXPHOS.^20^ Previous reports indicated that OXPHOS plays important roles not only in the maintenance of naive CD4^+^ T cells but also in the proliferation and effector function of helper and regulatory CD4^+^ T cells.^61^^,^^62^^,^^63^ Therefore, the observed slight decline of OXPHOS in *Cpox*-KO cells might be one of the causes of the decreased number of naive CD4^+^ T cells in *Cpox*-KO mice. On the other hand, we thought that the severe impairment of glycolysis observed in *Cpox*-KO cells contributed more crucially to their activation disorder, because the metabolic predominance shift from OXPHOS to glycolysis occurs as one of the key metabolic changes during T cell activation, as previously observed and shown by ourselves.^7^^,^^10^^,^^37^

OXPHOS-related gene expression was increased at the transcriptomic level, but OXPHOS activity was down. This is not surprising to see OXPHOS activity down in *Cpox*-KO T cells, considering the facts that heme-containing complexes should be non-functional without enough heme, some of OXPHOS-related protein abundance was actually decreased in *Cpox*-KO T cells and that heme-containing complexes IV abundance was reported to depend on the heme availability.^64^ OXPHOS-related gene expression was increased at the transcriptomic level when *Cpox* was deleted, suggesting that there is a feedback system that induces OXPHOS-related gene expression when OXPHOS activity is down. AMPK-PGC1a-OXPHOS-related gene axis may be part of this feedback system. AMP-activated protein kinase (AMPK) is activated when ATP levels are low, and it activates PGC1α, which increases the expression of OXPHOS-related genes.^65^ The importance of PGC1α is an emerging topic in the field of human chimeric antigen receptor (CAR) T cell therapy, as it is a key player in T cell metabolic reprogramming.^66^ Further analysis to reveal the relationship between heme metabolism and PGC1a will pave the way to improve the T cell fitness in CAR T cell therapy.

Glycolysis-related gene expression, including *Slc2a1* and *Hk2*, is regulated by IL-2-STAT5 signaling in CD4^+^ T cells.^42^ SLC2A1 imports glucose into CD4^+^ T cells, and HK2 is one of the rate-limiting enzymes in glycolysis. Here, we showed that *Slc2a1* and *Hk2* are the direct targets of p-STAT5. p-STAT5 also positively regulates *Il2rb*, the upstream factor of STAT5 signaling.^53^ Therefore, p-STAT5 drives a positive feedback loop in T cell activation, further amplifying their STAT5 signaling. In other words, STAT5 signaling activity should be tightly regulated for proper T cell activation. We found that the cell-intrinsic heme synthesis is required for the appropriate activation of STAT5 signaling, including glycolysis as a source of not only ATPs but also nucleotides through the pentose phosphate pathway.^67^

Heme regulates gene expression mainly through the repressive transcription factors BACH1 and BACH2 destabilization.^68^ Especially, BACH2 represses *Il2* expression itself in CD4^+^ T cells,^69^ and FOXP3-specific *Bach2*-KO mice progressed STAT5 phosphorylation and gene expression related to IL-2-STAT5 signaling.^70^ In line with this notion, we revealed key glycolysis-related genes, such as *Slc2a1* and *Hk2*, and some IL-2-STAT5 signaling-related genes, such as *Il2rb*, are targeted by both BACH2 and STAT5 at the common genomic loci, suggesting that BACH2 and STAT5 affect common genes by a mutually exclusive manner, as we had previously described the relationship between BACH transcription factors and transcription factor C/EBPα in myeloid cell progenitors.^71^^,^^72^ Because STAT5 signaling forms a positive feedback loop and promotes iron-heme metabolism as described earlier, the promotion of heme synthesis by IL-2-STAT5 signaling and the suppression of BACH2 lead to further enhancement of IL-2-STAT5 signaling.

In clinical settings, GVHD is one of the lethal complications in allogeneic hematopoietic stem cell transplantation. Although several new therapeutic agents like ruxolitinib, belumosudil, and ibrutinib have been approved for GVHD treatment in recent years,^73^^,^^74^^,^^75^ their outcomes in refractory severe GVHD patients are not adequately enough. Therefore, the identification of the new targets to treat GVHD is warranted. Previous studies showed that the impairment of both glycolysis and OXPHOS in T cells reduced cytokine production and CD4^+^ T cell proliferation to ameliorate GVHD.^76^ Here we show that *Cpox* deletion could ameliorate autoimmunity in several models, suggesting that the heme synthesis could be a target to treat autoimmune diseases, including GVHD, as previously described in other animal species.^77^^,^^78^

To the extent of our knowledge, overt lymphopenia or immunodeficiency has not been reported in porphyria patients, which might be because most human cases retain partial enzymatic activity, and immune function has not been systematically evaluated. Iron deficiency, on the other hand, is the most prevalent nutritional deficiency affecting world-wide.^79^ However, it is unclear whether physiological changes in iron availability significantly affect naive CD4^+^ T cell activation in clinical settings.^80^ Therefore, further analysis is needed to determine whether an adaptive immune disorder could occur in patients with porphyria and/or iron deficiency.

In conclusion, we found that cell-intrinsic heme synthesis is crucial for the activation of naive CD4^+^ T cells, supporting IL-2-STAT5 signaling and glycolysis. T cell activation might be controlled by micronutrients through “mitochondria-glycolysis crosstalk” during the key energy metabolism switch, in which mitochondria regulate glycolysis—a concept beyond the conventional model. Furthermore, these findings may contribute to the development of new therapeutic strategies for autoimmune diseases and GVHD.

### Limitations of the study

We found that heme synthesis impairment declined glycolysis during naive CD4^+^ T cell activation via intervention of the STAT5 pathway. We also considered the potential contributions of other signaling, such as c-*myc*, mTORC1, and HIF1A, but found no supportive evidence of their contribution to the disorder of naive CD4^+^ T cell activation by *Cpox* depletion. However, our analysis of these factors was limited. Therefore, we have to admit that pathways other than the STAT5 pathway could also contribute to the heme-dependent naive CD4^+^ T cell activation. Although inhibition of two distinct enzymes in the heme synthesis produced similar defects in naive CD4^+^ T cell proliferation that were rescued by hemin supplementation at least to some extent, we did not quantify porphyrin pathway intermediates in this study. Since hemin supplementation can also rescue the potential accumulation of porphyrin pathway intermediates, we cannot rule out the possibility that alterations in their accumulation also contributed to the observed phenotypes. Therefore, further study of the role of porphyrin pathway intermediates in naive CD4^+^ T cell proliferation, and further verification of our findings using additional KO models (e.g., establishing and analyzing the *Alas1/Alas2* conditional double-KO model) are needed in the future. In our transplantation experiments using irradiation, tissue damage-induced heme could affect our results as damage-associated molecular patterns (DAMPs).^81^ Although free heme is immediately trapped by heme scavenger proteins, such as hemopexin,^58^^,^^59^ and control or *Cpox*-KO donor T cells were transferred into identically irradiated recipients, thoughtful attention should be paid to interpret our results. Although we concluded that the heme synthesis could be a new therapeutic target for autoimmune diseases, including GVHD, we could not confirm this notion by using heme synthesis inhibitors due to the lack of available heme synthesis inhibitors *in vivo*. One of the concerns of using a heme synthesis inhibitor is its adverse effects. Notably, the impact of *Cpox* deletion in naive CD4^+^ T cells is limited, suggesting that the demand for heme synthesis increases when they are overactivated. Therefore, there might be a therapeutic window of heme synthesis inhibitors to suppress over-activating T cells without affecting non-activating T cells. These points should be revealed in the future.

## Resource availability

### Lead contact

Requests for further information, resources, and reagents should be directed to and will be fulfilled by the lead contact, Hiroki Kato (hiroki.kato.a5@tohoku.ac.jp).

### Materials availability

Unique materials generated in this study are available upon reasonable request from the lead contact with a completed materials transfer agreement.

### Data and code availability

• RNA sequence data generated in this study are publicly available at the Gene Expression Omnibus (GEO) under accession number GSE305952.
• This paper does not report original code.
• Any additional information required to reanalyze the data reported in this paper is available from the lead contact upon request.

## Acknowledgments

We thank all members of the Department of Hematology, Tohoku University Graduate School of Medicine, for their helpful support and discussions. We also thank the staff of the Biomedical Research Core of Tohoku University Graduate School of Medicine for their technical support. SFC was provided by SBI Pharmaceuticals. This study was supported by 10.13039/501100001691JSPS KAKENHI grant no. 24K02476 to H. Kato and H.H.

## Author contributions

Conceptualization, K.S., H. Kato, and H.H.; data curation, K.S., R.S., and H. Kato; funding acquisition, H. Kato and H.H.; analysis and investigation, K.S., H. Kato, Y.Y., S.S., D.M., K.H., N.M., Y.T., H. Komatsu, T.W., D.K., H. Kashiwagi, and F.W.; methodology, Y.Y., H. Kato, and F.W.; project administration, K.S. and H. Kato; resources, Y.Y. and S.S.; software, K.S. and H. Kato; supervision, K.O., S.H., Y.O., T.F., N.F., and H.H.; validation, K.S.; visualization, K.S.; writing – original draft, K.S.; writing – review and editing, all authors.

## Declaration of interests

The authors declare no competing interests.

## STAR★Methods

### Key resources table

**Table:** 

REAGENT or RESOURCE	SOURCE	IDENTIFIER
**Antibodies**		

FITC anti-mouse CD279 (PD-1) (Rat, Clone 29F.1A12)	BioLegend	Cat # 135213; RRID: AB_10689633
FITC anti-mouse CD3 epsilon (American Hamster, Clone 145-2C11)	BioLegend	Cat # 100306; RRID: AB_312670
FITC anti-mouse CD44 (Rat, Clone IM7)	BD Biosciences	Cat # 553133; RRID: AB_2076224
FITC anti-mouse NK-1.1 (Mouse, Clone PK136)	BioLegend	Cat #108706; RRID: AB_313393
PE anti-mouse CD25 (Rat, Clone PC61.5)	Tonbo biosciences	Cat #50–0251, RRID: AB_2621757
PE anti-mouse CD366 (Tim-3) (Rat, Clone RMT3-23)	BioLegend	Cat # 119703, RRID: AB_345377
PE anti-mouse CD45.1 (mouse, Clone A20)	BioLegend	Cat #110707, RRID: AB_313496
PE anti-mouse CD8a (Rat, Clone 53-6.7)	BD Biosciences	Cat #553033, RRID: AB_394571
PE anti-mouse anti-S6 (pS240) (Mouse, Clone N4-41)	BD Biosciences	Cat # 560430, RRID: AB_2572667
PE STAT5 (Mouse, Clone A-9)	Santa Cruz Biotechnology	Cat # sc-74442 PE, RRID: AB_1129711
PE/Cyanine7 anti-mouse CD62L (Rat, Clone MEL-14)	BioLegend	Cat # 104418, RRID: AB_313103
PE/Cyanine7 anti-mouse Ly6G/Ly6C (Gr1) (Rat, Clone RB6-8C5)	Thermo Fisher Scientific	Cat #25–5931081, RRID: AB_469662
PerCP/Cyanine5.5 anti-mouse CD223 (LAG-3) (Rat, Clone C9B7W)	BioLegend	Cat # 125211, RRID: AB_2561516
PerCP/Cyanine5.5 anti-mouse/human CD44 (Rat, Clone IM7)	BioLegend	Cat # 103031, RRID: AB_2076206
Alexa Fluor(R) 488 Anti-STAT5 (pY694) (Mouse, Clone 47/STAT5(pY694))	BD Biosciences	Cat # 612598, RRID: AB_399881
Alexa Fluor(R) 700 anti-mouse CD25 (Rat, Clone PC61)	BioLegend	Cat #102024, RRID: AB_493709
Alexa Fluor(R) 700 anti-mouse TER-119 (Rat, Clone TER-119)	BioLegend	Cat # 116220, RRID: AB_528963
APC anti-mouse/human CD44 (Rat, Clone IM7)	BioLegend	Cat # 103012, RRID: AB_312963
APC anti-mouse CD45.2 (Mouse, Clone 104)	BioLegend	Cat #109814, RRID: AB_389211)
APC anti-human/mouse CD45R (B220) (Rat, Clone RA3-6B2)	Tonbo biosciences	Cat # 20–0452, RRID: AB_2621574
APC/Cyanine7 anti-mouse CD4 (Rat, Clone RM4-5)	BioLegend	Cat # 100526, RRID: AB_312727
APC-H7 anti-mouse CD117 (Rat, Clone 2B8)	BD Biosciences	Cat # 560185, RRID: AB_1645231
Anti-mouse CD16/CD32 (Fc Block) (Rat, Clone 2.4G2)	BD Biosciences	Cat # 553142, RRID: AB_394657
Biotin anti-mouse/human CD11b (Rat, Clone M1/70)	BioLegend	Cat # 101204, RRID: AB_312787
Biotin anti-mouse CD11c (N/A, Clone N418)	Tonbo biosciences	Cat # 30–0114, RRID: AB_2621640
Biotin anti-mouse CD4 (Rat, Clone GK1.5)	BD Biosciences	Cat # 553728, RRID: AB_395012
Biotin anti-mouse CD45R/B220 (Rat, Clone RA3-6B2)	BD Biosciences	Cat # 553086, RRID: AB_394616
Biotin anti-mouse CD8a (Rat, Clone 53-6.7)	BD Biosciences	Cat # 553029, RRID: AB_394567
Biotin anti-mouse NK1.1 (Mouse, Clone PK136)	Tonbo biosciences	Cat # 30–5941, RRID: AB_2621653
Biotin anti-mouse TCR beta chain (American Hamster, Clone H57-597)	BioLegend	Cat # 109204, RRID: AB_313427
Biotin anti-mouse TCR gamma/delta (American Hamster, Clone GL3)	BioLegend	Cat # 118103, RRID: AB_313827
Biotin anti-mouse TER-119 (Rat, Clone TER-119)	BD Biosciences	Cat # 553672, RRID: AB_394985
ACTIN (I-19) sc-1616	Santa Cruz Biothecnology	Cat #G3012, RRID: AB_630836
HIF-1alpha (D2U3T) Rabbit mAb	Cell Signaling Technology	Cat # 14179, RRID: AB_2622225
Dnk pAb to Goat IgG (HRP)	abcam	Cat # ab97110, RRID: AB_10679463
Goat pAb to Rb IgG (HRP)	abcam	Cat # ab6721, RRID: AB_955447

**Chemicals, peptides, and recombinant proteins**		

Phosflow Fix Buffer I	BD Biosciences	Cat # 557870
Phosflow Perm Buffer III	BD Biosciences	Cat # 558050
Stain Buffer (FBS)	BD Biosciences	Cat # 554656
Cell Trace Violet	Thermo Fisher Scientific	Cat #C34557
Fixable Viability Stain 510	BD Biosciences	Cat # 564406
DAPI Solution	BD Biosciences	Cat # 564907
MitoTracker DeepRed FM	Thermo Fisher Scientific	Cat #M22426
MitoTracker Green FM	Thermo Fisher Scientific	Cat #M7514
UltraPure 0.5 M EDTA, pH 8.0	Thermo Fisher Scientific	Cat # 15575–038
Bovine Serum Albumin	Sigma	Cat # A2153
TexMACS Medium	Milteny Biotec	Cat # 130–097-196
2-Mercaptoethanol	Thermo Fisher Scientific	Cat # 21985023
Fetal bovine serum	SERENA	Cat # S-FBS-NL-015
Penicillin/Streptomycin	Sigma	Cat #P4333-100 ML
Succinyl acetone	Sigma	Cat #D1415
Sodium ferrous citrate	SBI Pharmaceuticals	N/A
Hemin	Sigma	Cat #H9039
Hemin	Tokyo Chemical Industry	Cat #H0008
5 mol/L Sodium Hydroxide Solution (5N)	KANTO CHEMICAL	Cat # 37844–08
IL-2	Miltenyi Biotec	Cat # 130–130-331
SuperScriptIV VILO Master Mix	Thermo Fisher Scientific	Cat # 1175650
ACK Lysis buffer	Thermo Fisher Scientific	Cat # A1049201
XF RPMI Assay Medium Pak	Agilent	Cat # 103681–100
Cell-Tak	Corning	Cat # 354240
FluxPak-XFe96/XE Pro Assay Pak	Agilent	Cat # 103792–100
QIAzol Lysis Reagent	QIAGEN	Cat # 79306
10% Formalin Neutral Buffer Solution	FUJIFILM Wako Pure Chemical	Cat # 062–01661
RIPA Buffer	Cell Signaling Technology	Cat # 9806S
Halt Protease & Phosphatase Inhibitor Cocktail	Thermo Fisher Scientific	Cat # 1861281
Total OXPHOS Rodent WB antibody cocktail	Abcam	Cat # ab110413
NuPAGE LDS Sample Buffer	Thermo Fisher Scientific	Cat # 2466927
Mini-PROTEAN TGX Gels 4–15%, 10 well comb, 30 μL	BIO-RAD	Cat # 4561083
Trans-Blot Turbo Transfer Membrane-mini	BIO-RAD	Cat # 1704156
Pierce ECL Plus Western Blotting Substrate	Thermo Fisher Scientific	Cat # 1610373

**Critical commercial assays**		

APC BrdU Flow Kit	BD Biosciences	Cat # 552598
FITC Annevin V Apoptosis Detection Kit with 7-AAD	BioLegend	Cat # 640922
APC Annexin V Apoptosis Detection Kit with 7-AAD	BioLegend	Cat # 640930
Naive CD4^+^ T cell isolation kit, mouse	Milteny Biotec	Cat # 130–104-453
CD4 (L3T4) Microbeads, mouse	Milteny Biotec	Cat # 130–117-043
T cell Activation/Expansion kit, mouse	Milteny Biotec	Cat # 130–093-627
Rneasy Plus Micro Kit	Qiagen	Cat # 74034
QuantiTect SYBR Green PCR Kit	Thermo Fisher Scientific	Cat # 204143
miRNeasy Micro kit	Qiagen	Cat # 217084
SMART-Seq HT Kit	Takara Bio	Cat # 634439
Seahorse XF T cell Metabolic Profiling Kit	Agilent	Cat # 103772–100
TC20 Automated Cell Counter	BIO-RAD	Cat # 145–0101
FACSAria II	BD Biosciences	N/A
NovaSea X Plus	Illumina	N/A
LCMS8500	Simazu	N/A
CFX Duet Real-Time PCR System	BIO-RAD	N/A
CFX96 Real-Time System	BIO-RAD	N/A

**Deposited data**		

Bulk RNA-seq data	This paper	GEO: GSE305952
Published BACH2 ChIP-seq data	Igarashi et al.^46^	GEO: GSE45975
Published BACH2 ChIP-seq data	Roychoudhuri et al.^47^	GEO: GSE126800
Published BACH2 ChIP-seq data	Sidwell et al.^48^	GEO: GSE127695
Published STAT5 ChIP-seq data	Grant et al.^49^	GEO: GSE207265
Published RNA-seq data	Geng et al.^50^	GEO: GSE123350

**Experimental models: Organisms/strains**		

B6/J	Jackson Laboratory Japan	Cat # 000664, RRID: IMSR_JAX:000664
B6.Cg-Tg(Cd4-cre)1Cwi/BfluJ	Jackson Laboratory Japan	Cat # 022071, RRID: ISMR_JAX:022071
B6.Cg-Rag2tm1.1Cgn/J	Jackson Laboratory Japan	Cat #008449, RRID: IMSR_JAX:008449
B6.SJL-Ptprca Pepcb/BoyJ	Jackson Laboratory Japan	Cat # 002014, RRID: IMSR_JAX:002014
BALB/cJ	Jackson Laboratory Japan	Cat # 000651, RRID: IMSR_JAX:000651
Cpox_A10	International Mouse Phenotyping Consortium	N/A

**Oligonucleotides**		

See Table S3 for qPCR Primer List	N/A	N/A

**Software and algorithms**		

FlowJo software 7.6.5 or 10.10.0	BD Biosciences	N/A
R 4.4.1	The R Project	N/A
iDEP 0.96	South Dakota State University	https://bioinformatics.sdstate.edu/idep96/
HOMER	Benner Lab	N/A
GraphPad Prism 10.6.1	Dotmatics	N/A
GSEA 4.3.3	BROAD Institute	N/A
IGV 2.18.4	BROAD Institute	N/A
DAVID	National Institute of Health	https://davidbioinformatics.nih.gov
ImageJ (Java 1.8.0_345)	National Institute of Health	https://imagej.net/ij/index.html
cellSens Standard 1.6	OLYMPUS	N/A

### Experimental model and study participant details

#### Mice

All mice were bred and housed under pathogen-free conditions in the Institute for Animal Experimentation in Tohoku University Graduate School of Medicine. C57BL/6 (B6; CD45.2^+^), BALB/c, Rag2-deficient, and CD4-Cre mice were purchased from Jackson Laboratory Japan. *Cpox*^f/f^ mice generated by inserting *loxP* sites flanking exon 2–4 were provided by the International Mouse Phenotyping Consortium.^82^ CB6F1 mice were bred from B6 and BALB/c mates. CD45.1^+^ mice for adoptive T cell transfer were maintained as homozygous populations. All mice between five to eight weeks of age were used for all experiments. RNA-seq analysis was performed using cells derived from only female mice. Sex-specific differences were not systematically evaluated in the other experiments, which may limit the generalizability of our findings across sexes. The Institutional Animal Care and Use Committee of Tohoku University approved all animal experiments (approval no. 2020MdA-111–08).

### Method details

#### Flow cytometry and cell sorting

The thymus and spleen were gently and mechanically dispersed by rubbing between two slide glasses and filtered with 70 μm cell strainers (Falcon). Erythroid cells in the thymus were lysed by ACK lysis buffer (Gibco). For cell sorting, erythroid cells in the spleen were also lysed by ACK lysis buffer. When extracellular staining was performed, the FcR blocking reagent was used to prevent non-antigen-specific binding. Extracellular antibodies were shown in key resources table. For cell trace violet staining, cells were incubated in the dark and at 37°C for 20 min. Culture medium or phosphate-buffered saline (PBS) was added, and the incubation was continued under the same conditions for 5 min.

For intracellular staining, dead cells were stained with Fixable Viability Stain 510 (BD Biosciences) for 15 min in the dark before extracellular staining.

For apoptosis assay, FITC or PE AnnexinV Apoptosis Kit (Biolegend) was used after extracellular staining as manufacturer’s protocol.

For cell cycle assay, APC BrDU kit (BD Biosciences) was used. *In vivo* assay, we administered BrdU intraperitoneally 1 h before sacrifice. *In vitro* assay, we added BrdU to culture medium at the terminal concentration of 10 μM then incubated for 90 min in the dark at 37°C under 5% CO_2_. Then we added extracellular staining, fixation and permeabilization as manufacturer’s protocol.

For phosphorylated flow cytometry, we fixed and permeabilized cells using Phosflow Fix Buffer I (BD Biosciences) and Phosflow Perm Buffer III (BD Biosciences) after dead cell staining. After permeabilization, we added extracellular and intracellular staining antibodies and incubated in the dark at room temperature for 1 h.

For mitotracker staining, we used MitoTracker Green FM and Deep Red FM (Thermo Fisher Scientific). We incubated cell suspension with these reagents for 40 min in the dark at 37°C under 5% CO_2_. After that, we added extracellular staining.

These samples were analyzed using a FACS Aria II (BD Biosciences), and quantitative data analysis was performed using FlowJo software (BD Biosciences).

#### Quantitative real-time PCR (qPCR) analysis

Total RNA was extracted using the RNeasy Plus Micro Kit (QIAGEN), then complementary DNA was synthesized by the SuperScript IV VILO Master Mix (Thermo Fisher Scientific). The QuantiTect SYBR Green PCR Kit (Thermo Fisher Scientific) was used for qPCR. Results from each experiment were normalized by the mRNA expression of *Actb*. The list of primer sequences is shown in Table S3.

Each sample was collected as follows. Cells were collected by flow cytometric sorting from the thymus or spleen in Figure 1A according to Table S1. In Figures 2A and S6G, bulk naive or activated CD4^+^ cells were collected just after magnetic cell sorting or after *in vitro* activation on the indicated days.

#### *In vitro* proliferation of naive CD4^+^ T cells

Naive CD4^+^ T cell Isolation Kit (Miltenyi Biotec) was used for erythrocyte-lysed splenic cell suspensions according to the manufacturer’s protocol. Then, naive CD4^+^ T cells were cultured at a concentration of 1×10^6^/mL in TexMACS medium (Miltenyi Biotec) supplemented with 10% FBS, 1% penicillin/streptomycin, 0.018% 2-mercaptoethanol, and 25 U/mL IL-2 (Miltenyi Biotec) at 37°C with 5% CO_2_. Hemin (Sigma) was added to the culture medium on day 0 at a concentration of 0–30 μM, dissolved in 0.1N NaOH, for the rescue experiments. SFC (SBI) was added to the culture on day 0 at a concentration of 0–30 μM. Succinyl acetone (Sigma) was utilized at a concentration of 0–1.0 mM for heme synthesis inhibition experiments. Cells were activated by CD3 and CD28 stimulation using the T cell Activation/Expansion Kit (Miltenyi Biotec) according to the manufacturer’s protocol from Day 0. On day 2, cells were gently pipetted, split 1:2, and added the same amount of fresh culture medium. This culture condition was used as the *in vitro* activation protocol throughout this study. We assessed the cell concentration in wells using a cell counter (Bio-Rad) for the proliferation assay. The proliferation ratio was calculated as the cell count on the indicated day divided by the cell count on Day 0 in each group.

#### Measurement of total heme by mass spectrometry

The total heme (hemin) content was measured by mass spectrometry according to a previously described method with modification.^83^ Briefly, 0.4–1 × 10^6^ naive CD4^+^ (Day 0) or activated CD4^+^ (Day 2) T cells from wild type mice or 1 × 10^4^ activated CD4^+^ (Day 2) T cells from control or *Cpox*-KO mice were sorted as CD4^+^ cells using FACS Aria II (BD Biosciences). Day 0 cells were collected immediately after magnetic isolation of naive CD4^+^ T cells, whereas Day 2 were collected two days after *in vitro* activation. Then these cells were suspended in 0.1 mL extraction buffer (acetonitrile: 2N HCl (8:2)). After a brief sonication and vortexing, cell lysate was centrifuged at 10,000 g for 5 min, the supernatant, which contained total hemin, was subjected to mass spectrometry (Shimadzu). The peak corresponding to hemin was confirmed using an authentic standard purchased from TCI (Tokyo Chemical Industry, H0008). The area of the peak corresponding to hemin was normalized to the cell number, then calculated ratio between Day 0 to Day 2 or control to *Cpox*-KO.

#### Bulk RNA sequence

RNA was obtained from 1×10^4^ control or *Cpox*-KO CD4^+^ T cells before activation (Day 0; CD4^+^ T cells were sorted using FACS Aria II just after magnetic cell sorting for naive CD4^+^ T cells) and after activation (Day 2 and Day 4; CD4^+^ T cells were sorted using FACS Aria II on each day). Cells were lysed by QIAzol Lysis Reagent (Qiagen) then extracted by miRNeasy Micro kit (Qiagen). Full-length cDNA Library was prepared using an SMART-Seq HT Kit (Takara Bio). Data were processed by NovaSeq X Plus (Illumina). Sequence reads were trimmed for adaptor sequences and low-quality bases using fastp.^84^ Trimmed sequence reads were mapped to the mouse (mm10) reference genome using HISAT2.^85^ Read count extraction and normalization were performed using StringTie.^86^^,^^87^^,^^88^^,^^89^ We analyzed these data using iDEP.96 (South Dakota State University), GSEA 4.3.3 software (BROAD Institute), DAVID (NIH), IGV (BROAD Institute) and HOMER (Benner Lab). Data are available at the Gene Expression Omnibus (GEO) under accession number GSE305952.

#### Metabolic assays

Naive CD4^+^ T cells were isolated by magnetic cell sorting. Day 0 naive CD4^+^ T cells were collected immediately after magnetic isolation, whereas Day 2 activated CD4^+^ T cells were collected two days after *in vitro* activation as described above. Naive or activated CD4^+^ T cells were prepared at a concentration of 4×10^6^ cells/mL in assay medium (Agilent, XF RPMI medium with 10 mM glucose, 1 mM sodium pyruvate and 2 mM glutamate). Cells were seeded onto Cel-Tak (Corning) coated 96-well assay microplates (Agilent), then centrifuged 200 *g* at room temperature for 1 min twice. After that, cells were incubated at 37°C in room air for 60 min. We assessed metabolism by XF T cell metabolic profiling kit (Agilent) and Seahorse XF Pro extracellular flux analyzer (Agilent).

#### Western blot analysis

Naive CD4^+^ T cells were isolated by magnetic cell sorting and activated *in vitro* as described above. Bulk *in vitro* activated CD4^+^ T cells were collected at 1-2×10^6^ cells per sample. Cells were lysed by RIPA buffer (Cell Signaling Technology) according to the manufacturer’s instructions. Protease phosphatase inhibitor (Thermo Fisher Scientific) was added to prevent protein degradation and stabilize phosphorylation, then samples were vortexed and incubated on ice for 5 min. After sonication, samples were centrifuged at 14,000 g at 4°C for 10 min. Supernatant was collected and NuPAGE LDS sample buffer (Thermo Fisher Scientific) was added to prepare protein samples for electrophoresis. The samples were boiled at 95°C for 10 min. The samples were loaded to wells of Mini-PROTEAN TGX gels (Bio-RAD), then run electrophoresis. Protein was transferred from gels to membranes by Trans-Blot Turbo Transfer System (Bio-RAD). The membrane was blocked in 5% milk at room temperature for 1 h. After blocking, they were incubated with primary antibodies at room temperature for 2 h in following concentrations: HIF-1 alpha (Cell Signaling Technology, 1/1000), total OXPHOS rodent antibody cocktail (Abcam, 1/1000) and ACTIN (Santa Cruz Biotechnology, 1/1000). After incubation, the membranes were washed, then incubated with secondary antibodies at room temperature for 1 h in following concentrations: anti-Rabbit (abcam, 1/3000) and anti-Goat (abcam, 1/10000). Finally, the images were measured using Pierce ECL Plus Western Blotting Substrate (Thermo Fisher Scientific) and FUSION SOLO.7S (VILBER).

#### Adoptive T cell transfer

Adoptive T cell transfer experiments were performed using two different types of recipients. Splenic 1×10^6^ naive CD4^+^ T cells magnetically isolated from 5–8 weeks old control or *Cpox*-KO mice (CD45.2^+^) were transferred to one-day-before sub-lethally irradiated (5.0 Gy) sex matched 5–8 weeks old CD45.1^+^ mice. 5×10^5^ naive CD4^+^ T cells from 5–8 weeks old control or *Cpox*-KO mice were transferred to sex matched 5–8 weeks old *Rag2*-KO mice. One week later, splenic T cells were analyzed in both models and two weeks later in the *Rag2*-KO model unless otherwise mentioned. For *Rag2*-KO models, body weight was measured every week until 8 weeks after transplantation. We conducted pathological and anatomical assessment 4 weeks after the transfer.

#### GVHD model

We obtained recipient CB6F1 mice from B6 and BALB/c mice mates. 6–8 weeks old recipient mice were lethally irradiated (8.5 Gy) one day before transplantation. 5 × 10^5^ CD4-depleted bone marrow cells using CD4 (L3T4) microbeads (Miltenyi Biotec) from control mice, and splenic 1.0 × 10^6^ naive CD4^+^ T cells obtained by magnetic cell sorting from control or *Cpox*-KO mice, which were 6–8 weeks old and sex matched, were transferred. We analyzed GVHD score as previously described,^90^ and survival twice a week. If the GVHD score reached seven or over, we ethically sacrificed the mice. We conducted pathological and anatomical assessment on day 14 after transplantation.

### Quantification and statistical analysis

Statistical analyses were performed using Excel (v16.87) or GraphPad Prism (v10.6.1). Comparisons between two groups were conducted using unpaired Student’s *t* test and three or more groups were by one-way ANOVA followed by multiple comparison tests (Tukey or Dunnett). For time-course experiments, two-way ANOVA, two-way repeated measures ANOVA or two-way mixed-effects measures ANOVA test to compare two or more groups was performed followed by multiple comparison tests (Tukey or Sidak) as appropriate.

Data were presented as mean ± standard deviation unless otherwise indicated. The sample size (n) represents the number of independent biological samples or animals as specified in the corresponding figure legends. Statistical details were also described in the corresponding figure legends. A *p*-value <0.05 was considered statistically significant (∗*p* < 0.05, ∗∗*p* < 0.01, ∗∗∗*p* < 0.001).
