# Supplementary material for: Cell intrinsic heme synthesis is required for the proper activation of naive CD4+ T cells
Source: iScience. 2026 Nov 4;29(11):118114. doi: 10.1016/j.isci.2026.118114 (PMC13753341; doi:10.1016/j.isci.2026.118114)
Supplement: Supplementary file 1 [file mmc1.pdf]

iScience, Volume *29*

Supplemental information

Cell intrinsic heme synthesis is required for the proper activation of naive $CD4^+$ T cells

Kazuki Sakurai, Hiroki Kato, Yan Yan, Sayaka Sano, Ryota Saito, Daigo Michimata, Kazuki Hashimoto, Naoya Morota, Yuya Tanaka, Hiroka Komatsu, Tatsuya Watanabe, Daichi Kiba, Hitomi Kashiwagi, Koichi Onodera, Shunsuke Hatta, Yasushi Onishi, Tohru Fujiwara, Takaaki Abe, Fan-Yan Wei, Noriko Fukuhara, and Hideo Harigae

SUPPLEMENTAL INFORMATION

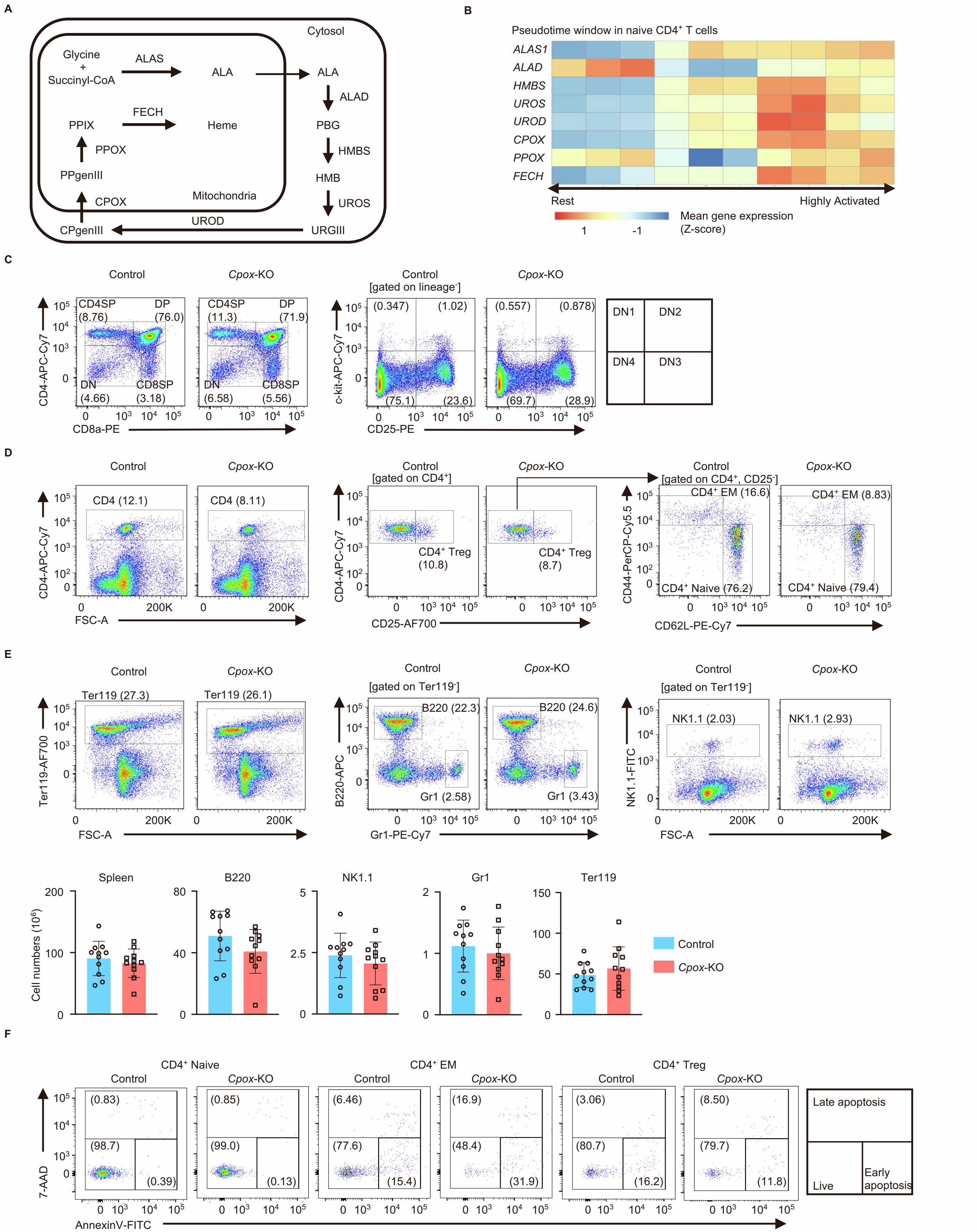
A
Cytosol
Glycine
+
Succinyl-CoA
ALAS
ALA
ALA
ALAD
PBG
HMBS
HMB
UROS
URGIII
UROD
CPgenIII
CPOX
PPgenIII
PPOX
PPIX
FECH
Heme
Mitochondria
B
Pseudotime window in naive CD4+ T cells
ALAS1
ALAD
HMBS
UROS
UROD
CPOX
PPOX
FECH
Rest
Highly Activated
Mean gene expression
(Z-score)
1
-1
C
Control
Cpox-KO
CD4SP (8.76)
DP (76.0)
DN (4.66)
CD8SP (3.18)
CD4SP (11.3)
DP (71.9)
DN (6.58)
CD8SP (5.56)
CD4-APC-Cy7
CD8a-PE
Control
[gated on lineage-]
Cpox-KO
(0.347)
(1.02)
(75.1)
(23.6)
(0.557)
(0.878)
(69.7)
(28.9)
c-kit-APC-Cy7
CD25-PE
DN1
DN2
DN4
DN3
D
Control
Cpox-KO
CD4 (12.1)
CD4 (8.11)
CD4-APC-Cy7
FSC-A
Control
[gated on CD4+]
Cpox-KO
CD4+ Treg (10.8)
CD4+ Treg (8.7)
CD4-APC-Cy7
CD25-AF700
Control
[gated on CD4+, CD25-]
Cpox-KO
CD4+ EM (16.6)
CD4+ Naive (76.2)
CD4+ EM (8.83)
CD4+ Naive (79.4)
CD44-PerCP-Cy5.5
CD62L-PE-Cy7
E
Control
Cpox-KO
Ter119 (27.3)
Ter119 (26.1)
Ter119-AF700
FSC-A
Control
[gated on Ter119-]
Cpox-KO
B220 (22.3)
Gr1 (2.58)
B220 (24.6)
Gr1 (3.43)
B220-APC
Gr1-PE-Cy7
Control
[gated on Ter119-]
Cpox-KO
NK1.1 (2.03)
NK1.1 (2.93)
NK1.1-FITC
FSC-A
Spleen
B220
NK1.1
Gr1
Ter119
Cell numbers (10^6)
Control
Cpox-KO
F
CD4+ Naive
CD4+ EM
CD4+ Treg
Control
Cpox-KO
(0.83)
(98.7)
(0.39)
(0.85)
(99.0)
(0.13)
(6.46)
(77.6)
(15.4)
(16.9)
(48.4)
(31.9)
(3.06)
(80.7)
(16.2)
(8.50)
(79.7)
(11.8)
7-AAD
AnnexinV-FITC
Late apoptosis
Live
Early apoptosis

Supplementary Figure 2

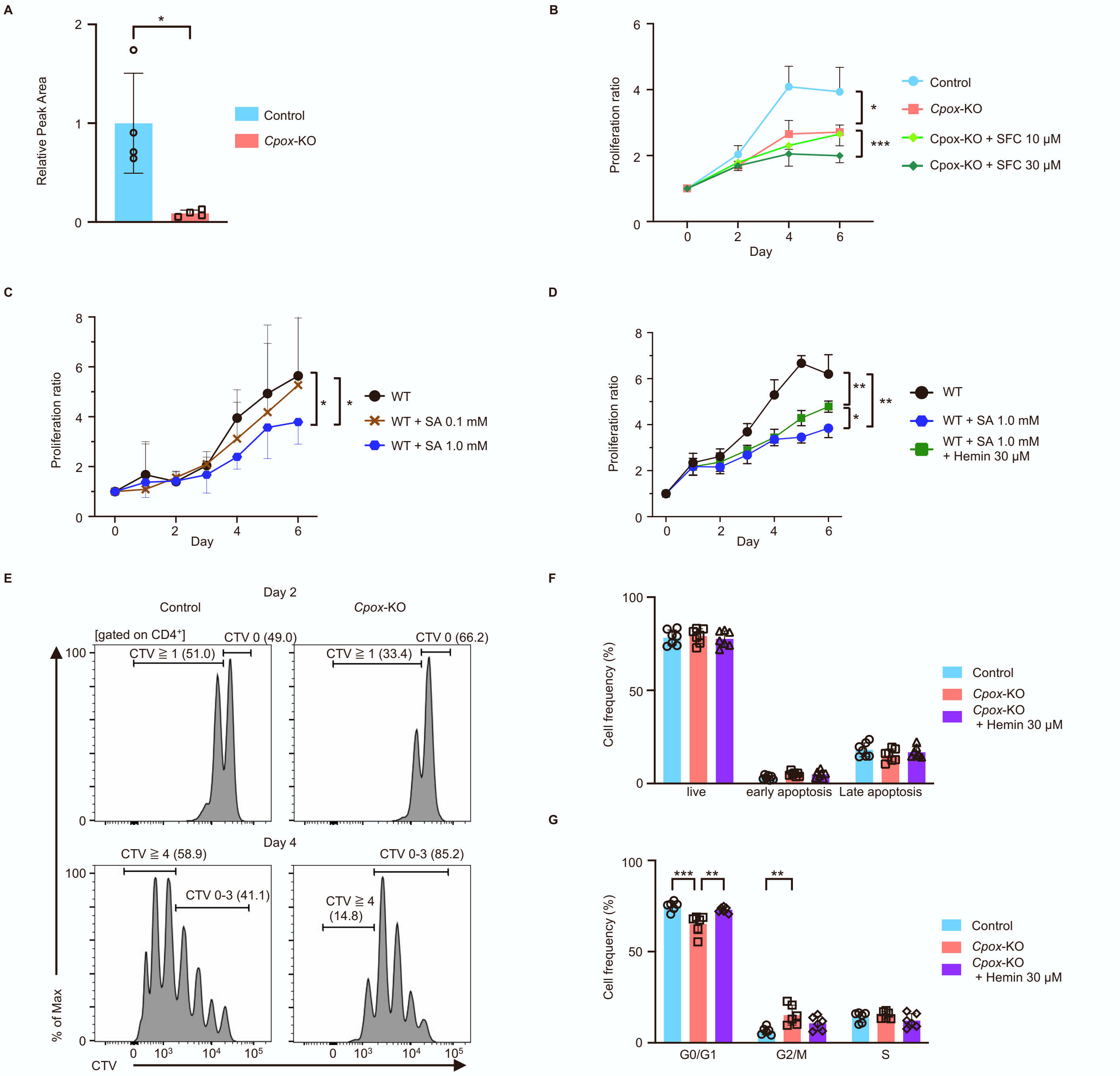

Supplementary Figure 3

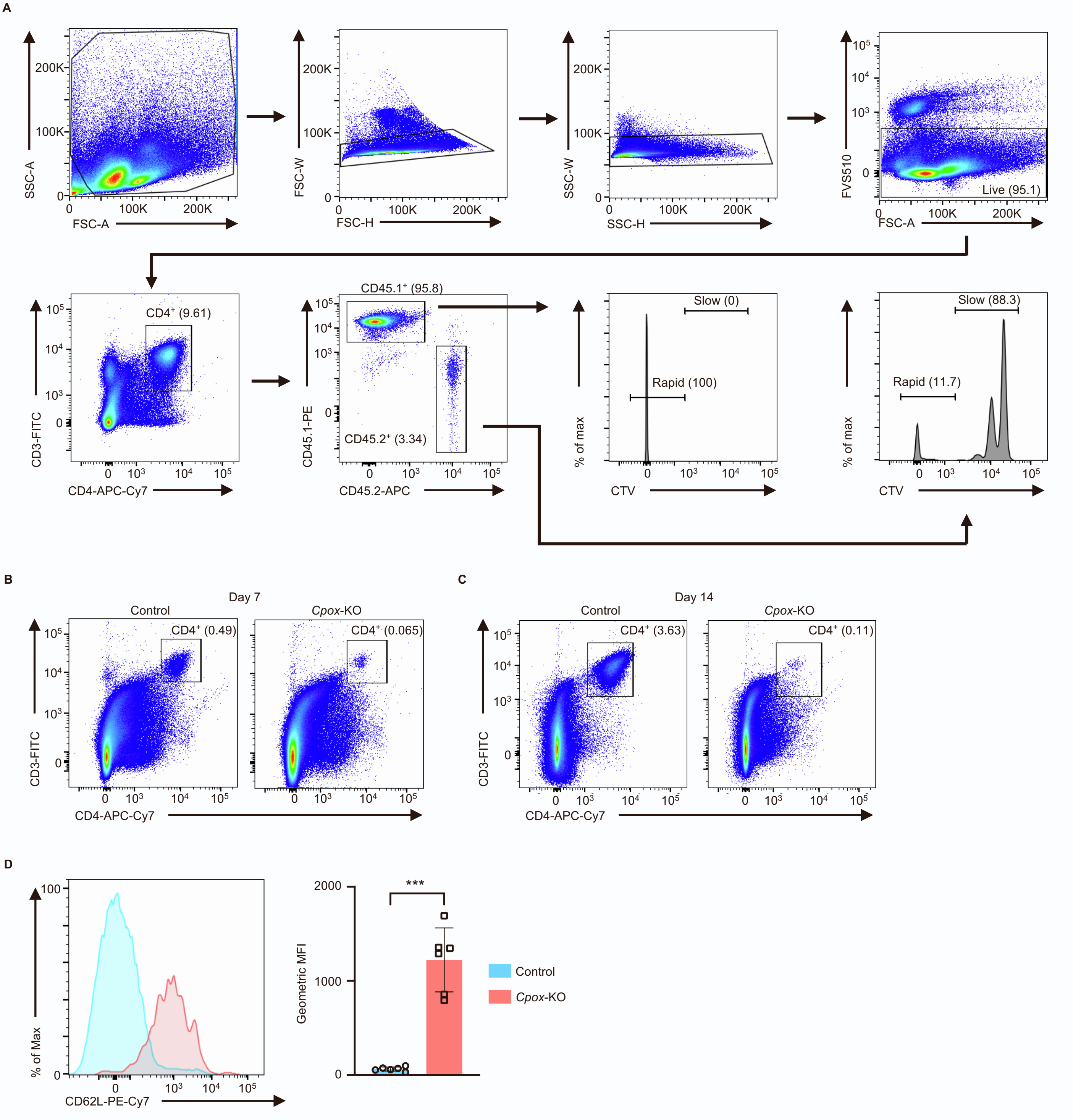

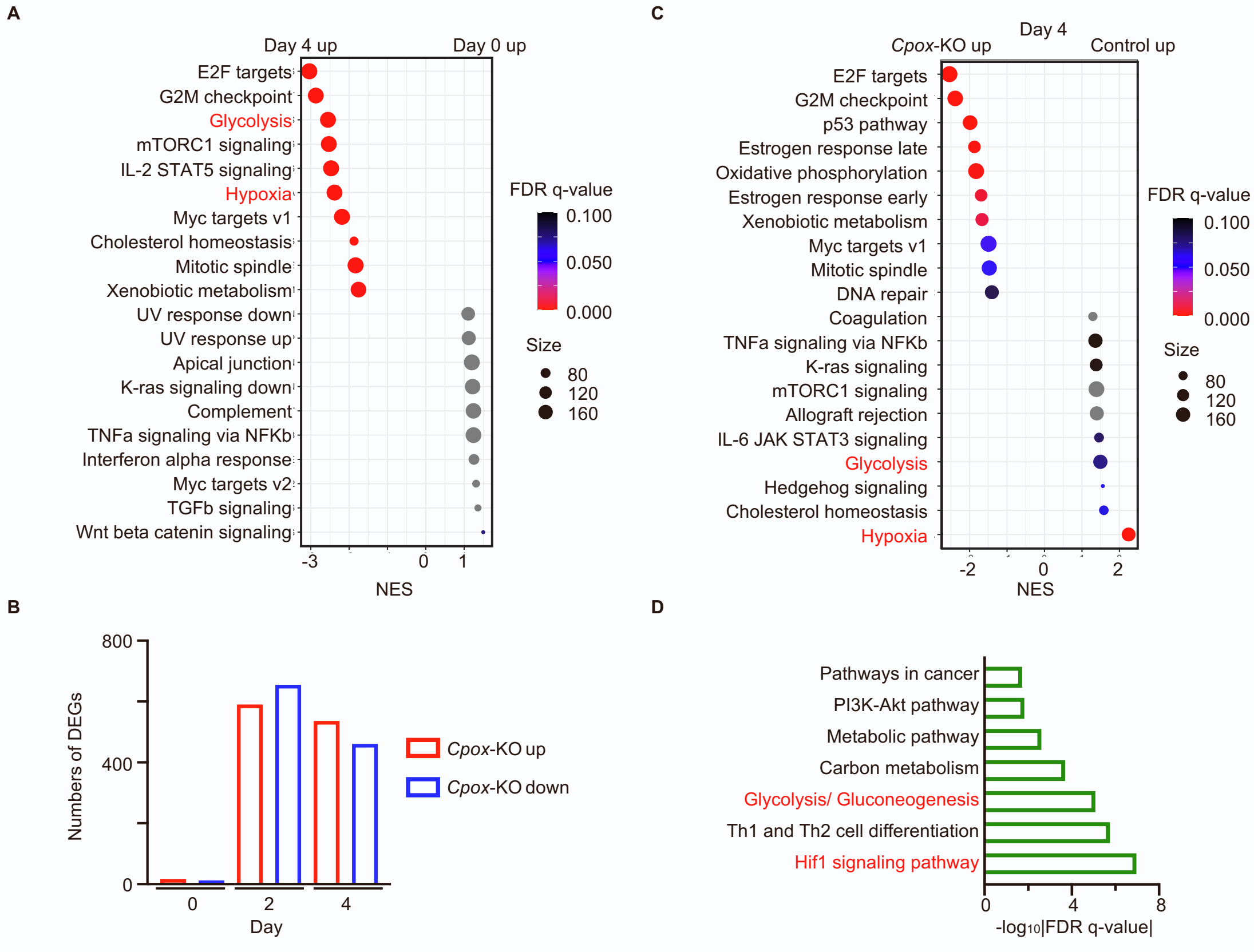
A
Day 4 up
Day 0 up
E2F targets
G2M checkpoint
Glycolysis
mTORC1 signaling
IL-2 STAT5 signaling
Hypoxia
Myc targets v1
Cholesterol homeostasis
Mitotic spindle
Xenobiotic metabolism
UV response down
UV response up
Apical junction
K-ras signaling down
Complement
TNFa signaling via NFKb
Interferon alpha response
Myc targets v2
TGFb signaling
Wnt beta catenin signaling
-3
0
1
NES
FDR q-value
0.100
0.050
0.000
Size
80
120
160
C
Day 4
Cpox-KO up
Control up
E2F targets
G2M checkpoint
p53 pathway
Estrogen response late
Oxidative phosphorylation
Estrogen response early
Xenobiotic metabolism
Myc targets v1
Mitotic spindle
DNA repair
Coagulation
TNFa signaling via NFKb
K-ras signaling
mTORC1 signaling
Allograft rejection
IL-6 JAK STAT3 signaling
Glycolysis
Hedgehog signaling
Cholesterol homeostasis
Hypoxia
-2
0
2
NES
FDR q-value
0.100
0.050
0.000
Size
80
120
160
B
800
400
0
Numbers of DEGs
Cpox-KO up
Cpox-KO down
0
2
4
Day
D
Pathways in cancer
PI3K-Akt pathway
Metabolic pathway
Carbon metabolism
Glycolysis/ Gluconeogenesis
Th1 and Th2 cell differentiation
Hif1 signaling pathway
0
4
8
-log10|FDR q-value|

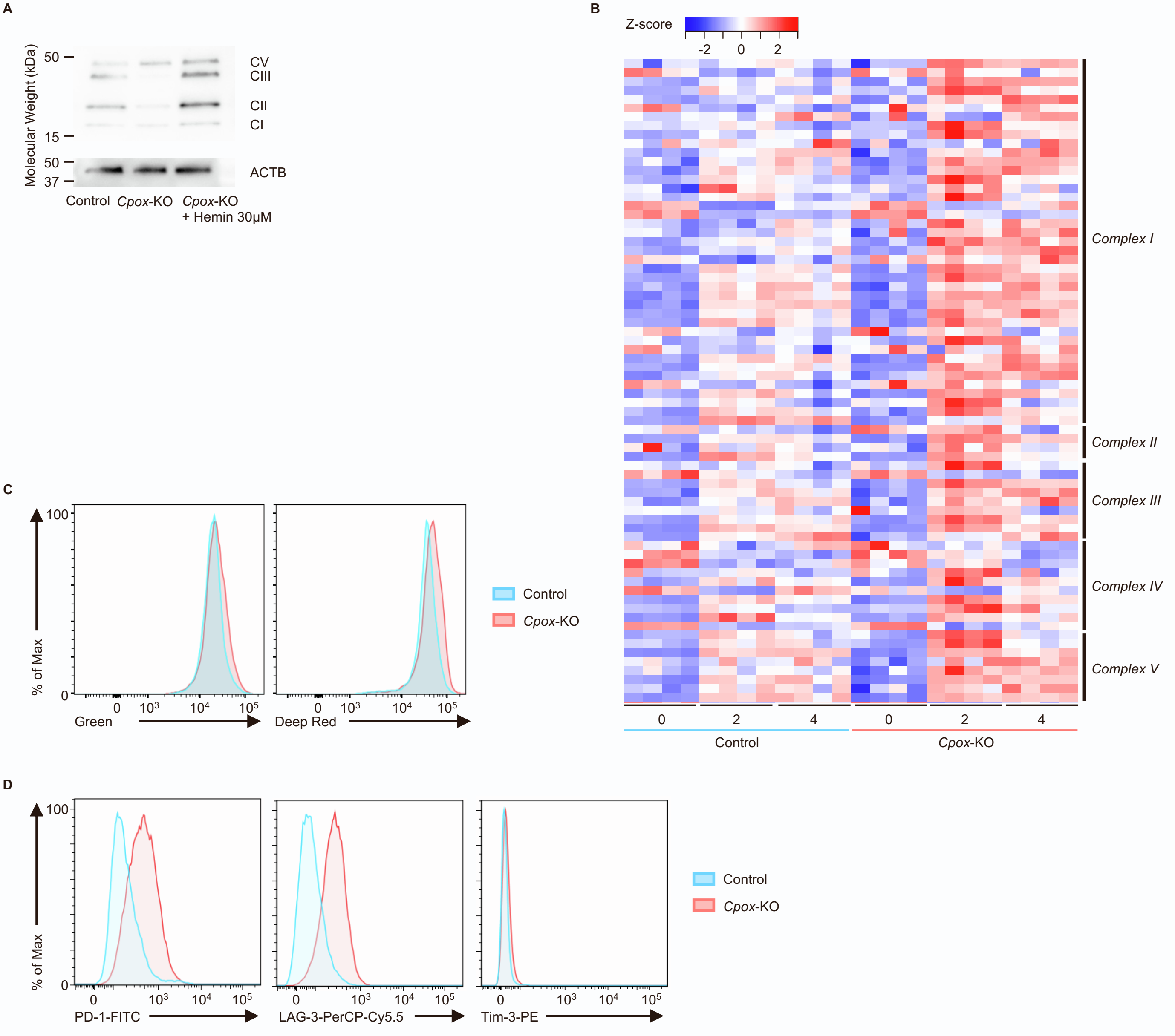
A
Molecular Weight (kDa)
50
15
50
37
CV
CIII
CII
CI
ACTB
Control
Cpox-KO
Cpox-KO + Hemin 30μM
B
Z-score
-2
0
2
Complex I
Complex II
Complex III
Complex IV
Complex V
0
2
4
Control
Cpox-KO
C
% of Max
100
Green
Deep Red
Control
Cpox-KO
D
PD-1-FITC
LAG-3-PerCP-Cy5.5
Tim-3-PE

Supplementary Figure 6

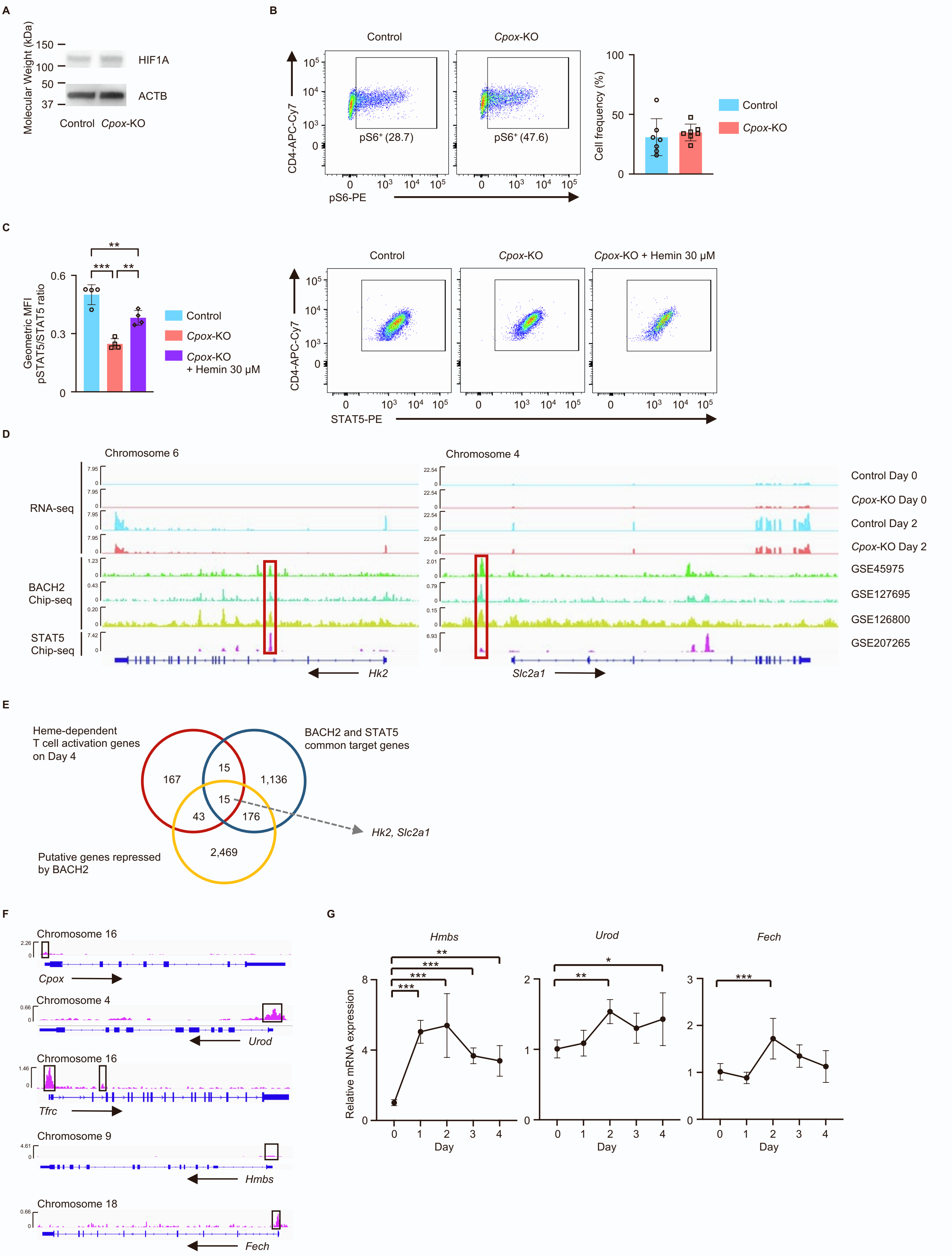

Supplementary Figure 7

A

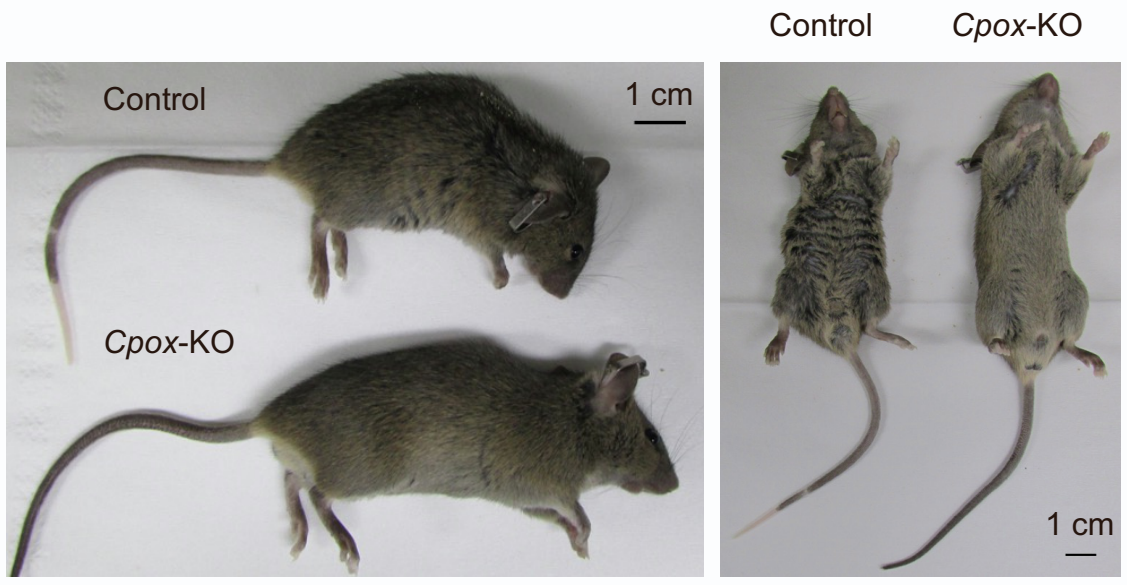

SUPPLEMENTAL FIGURE LEGENDS

Figure S1. The schema of heme synthesis, publicly available data, and representative flow cytometry plots, related to Figure 1

(A) The schema of the cell intrinsic heme synthesis.

(B) Pseudotime analysis of single-cell RNA sequence of heme synthetic enzymes in naive human CD4$^+$ T cell activation.

(C)-(E) Gating strategy of flow cytometry in the thymus (C), splenic CD4$^+$ T cells (D), and other splenocytes (E). Color bars showed the absolute numbers of indicated cells (n = 11 mice/group).

(F) Gating strategy of flow cytometry in apoptosis assay, related to Figure 1D.

Each dot represents one mouse/sample. Accumulated data from two or more experiments are shown as mean ± standard deviation. Statistical analysis was performed using two-tailed Student's t-test (*p < 0.05, **p < 0.01, ***p < 0.001).

Figure S2. The effect of succinyl acetone treatment for wild-type naive CD4$^+$ T cell proliferation, representative histograms of flow cytometry, apoptosis analysis, and cell cycle analysis, related to Figure 2

(A) Intracellular heme concentration of control or *Cpox*-KO in vitro activated CD4$^+$ T cells on Day 2 was assessed by mass spectrometry (n = 4 mice/group). To obtain these cells, we collected CD4$^+$ T cells by flow cytometric sorting as described in STAR Methods.

(B) Proliferation ratio of naive CD4$^+$ T cells with or without SFC addition (n = 6

mice/group).

(C) Proliferation ratio of naive $CD4^+$ T cells from wild-type mice with or without succinyl acetone (SA) (n = 6 mice/group).

(D) Proliferation ratio of naive $CD4^+$ T cells from wild-type mice with or without SA and hemin (n = 6 mice/group).

(E) Gating strategy of flow cytometry in cell trace violet (CTV) staining, related to Figure 2E. Figure 2F was also analyzed similarly.

(F) Apoptosis rates of in vitro activated $CD4^+$ T cells (Day 4) were analyzed by flow cytometry (n = 6 mice/group).

(G) Cell cycles of in vitro activated $CD4^+$ T cells (Day 4) were analyzed by flow cytometry (n = 7 mice/group).

Each dot represents one mouse/sample. Accumulated data from two or more experiments are shown as mean ± standard deviation. Statistical analysis was performed using two-tailed Student's t-test for (A) or two-way repeated measures ANOVA test followed by Sidak multiple comparison tests for proliferation ratio whose primary endpoint was on Day 6 (B-D) and one-way ANOVA test followed by Tukey (F, G) multiple comparison tests (*$p < 0.05$, **$p < 0.01$, ***$p < 0.001$).

Figure S3. Gating strategy and CD62L expression of flow cytometry of homeostatic lymphoproliferation models, related to Figure 3

(A) Gating strategy of flow cytometry in adoptive naive $CD4^+$ T cell transfer to $CD45.1^+$ mice related to Figure 3C. This figure showed the representative pictures from control mice but the same gates were used for analysis in both groups.

(B-C) Gating strategy of flow cytometry in adoptive naive $CD4^+$ T cell transfer to *Rag2*-KO mice related to Figure 3E. (B) Day 7, (C) Day 14.

(D) Representative histogram of CD62L expression by flow cytometry (left) and geometric mean fluorescent intensity of CD62L (right) of $CD4^+$ T cells in transferred *Rag-2* KO mice on Day 14 (n = 6 recipient mice/group). Each dot represents each mouse/sample. Accumulated data from two or more experiments are shown with the standard deviation. Statistical analysis was performed using two-tailed Student's t-test (*p < 0.05, **p < 0.01, ***p < 0.001).

Figure S4. Additional analysis of RNA-seq data, related to Figure 4

(A) Top and bottom 10 gene sets from GSEA mouse hallmark gene sets comparing Day 0 and Day 4 of control naive $CD4^+$ T cell activation.

(B) The number of differentially expressed genes (DEGs) between control and *Cpox*-KO cells at the indicated time points. DEGs were defined as $|\log_2 FC| > 0.585$ and FDR-q value < 0.1.

(C) Top and bottom 10 gene sets from GSEA mouse hallmark gene sets comparing control and *Cpox*-KO naive $CD4^+$ T cell activation on Day 4.

(D) DAVID analysis of genes, which was merged DEGs upregulated on Day 2 comparing Day 0 (control) and control comparing *Cpox*-KO (Day 2).

Figure S5. Gene expression profile of OXPHOS-related genes and histogram of flow cytometry, related to Figure 5

(A) Representative western blot analysis of OXPHOS-related protein (Complex I to III

and V) and ACTB in naive $CD4^+$ T cell activation on Day 2. Uncropped original images are shown in Data S1A.

(B) Gene expression profile of OXPHOS-related genes of control and *Cpox*-KO naive $CD4^+$ T cells during their activation (Day 0, Day 2, and Day 4).

(C) Representative histograms of mitotracker staining of green (left) and deep red (right), related to Figure 5C.

(D) Representative histograms of inhibitory receptors, which were considered as exhaustion markers; PD-1 (left), LAG-3 (middle), and Tim-3 (right), related to Figure 5D.

Figure S6. Western blot analysis, flow cytometry plot, merged analysis of ChIP-seq and RNA-seq data, gene tracks, and gene expression, related to Figure 6

(A) Representative western blot analysis of HIF1A and ACTB in naive $CD4^+$ T cell activation on Day 2. Uncropped original images are shown in Data S1B.

(B) Gating strategy of flow cytometry of phosphorylated S6 ($pS6^+$) (left) and $pS6^+$ cell frequency (right) of activated $CD4^+$ T cells (Day 2) (n = 7 mice). Accumulated data from two or more experiments are shown with the standard deviation.

(C) Relative MFI ratio of phosphorylated STAT5 to total STAT5 (n = 4 mice/group) (left) and gating strategy of flow cytometry of total $STAT5^+$ cell frequency (right) of activated $CD4^+$ T cells on Day 2. Accumulated data from two experiments are shown with the standard deviation.

(D) Gene tracks of *Hk2* and *Slc2a1*, including our RNA-seq data, previous BACH2 ChIP-seq data, and STAT5 ChIP-seq data.

(E) Merged figure of our RNA sequence data and previous data. The red ring shows

merged upregulated genes (|$\log_2$FC| > 0.585) of control Day 4 compared to control Day 0, and control cells compared to *Cpox*-KO cells (Day 4) using our RNA-seq data. The blue ring shows merged STAT5 ChIP-seq (GSE207265) and three BACH2 ChIP-seq data (GSE45975, GSE126800, and GSE127695). Genes that had common enrichment on the same genomic loci in all four ChIP-seq data were shown. The yellow ring shows upregulated genes (FC > 1.25) in *Bach2*-KO T cell versus wild-type T cell RNA-seq data (GSE123350). Upregulated genes (FC > 1.25) in *Bach2*-KO T cells compared to wild-type T cells were shown. Fifteen extracted genes were listed in Table S2B.

(F) Gene tracks of heme synthases and *Tfrc*, which had the genomic loci of STAT-5 enrichment (GSE207265).

(G) Relative mRNA expression of *Hmbs*, *Urod,* and *Fech* during wild-type naive $CD4^+$ T cell activation was analyzed by qPCR (n = 6 mice).

Each dot represents one mouse/sample (B, C). Accumulated data from two or more experiments are shown as mean ± standard deviation. Statistical analysis was performed using two-tailed Student's t-test (B), one-way ANOVA test followed by Tukey (C) and followed by Dunnett multiple comparison tests (G) (*p < 0.05, **p < 0.01, ***p < 0.001).

Figure S7. Representative GVHD mice picture, related to Figure 7

(A) The representative pictures of GVHD model mice on Day 14. The left pictures were mouse posture (upper: control cell transferred group, lower: *Cpox*-KO cell transferred group). The right pictures represented ventral fur (left; control cell transferred group, right side; *Cpox*-KO cell transferred group).

A

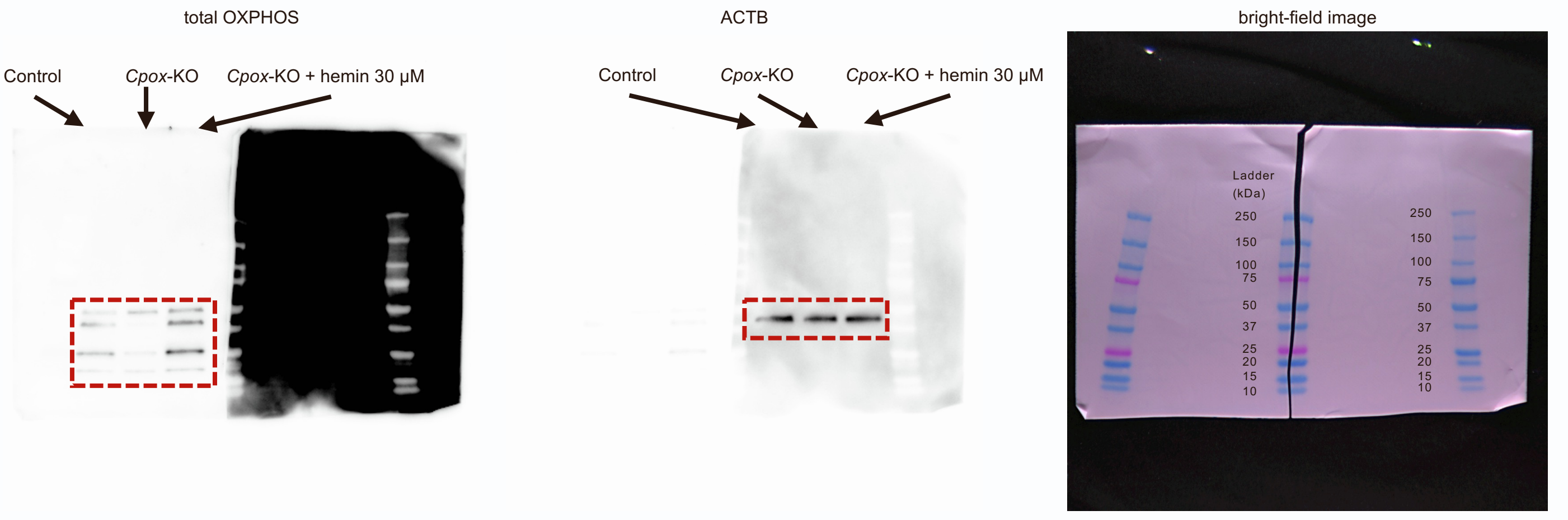
total OXPHOS
ACTB
bright-field image
Control
Cpox-KO
Cpox-KO + hemin 30 μM
Control
Cpox-KO
Cpox-KO + hemin 30 μM
Ladder
(kDa)
250
150
100
75
50
37
25
20
15
10
250
150
100
75
50
37
25
20
15
10

B

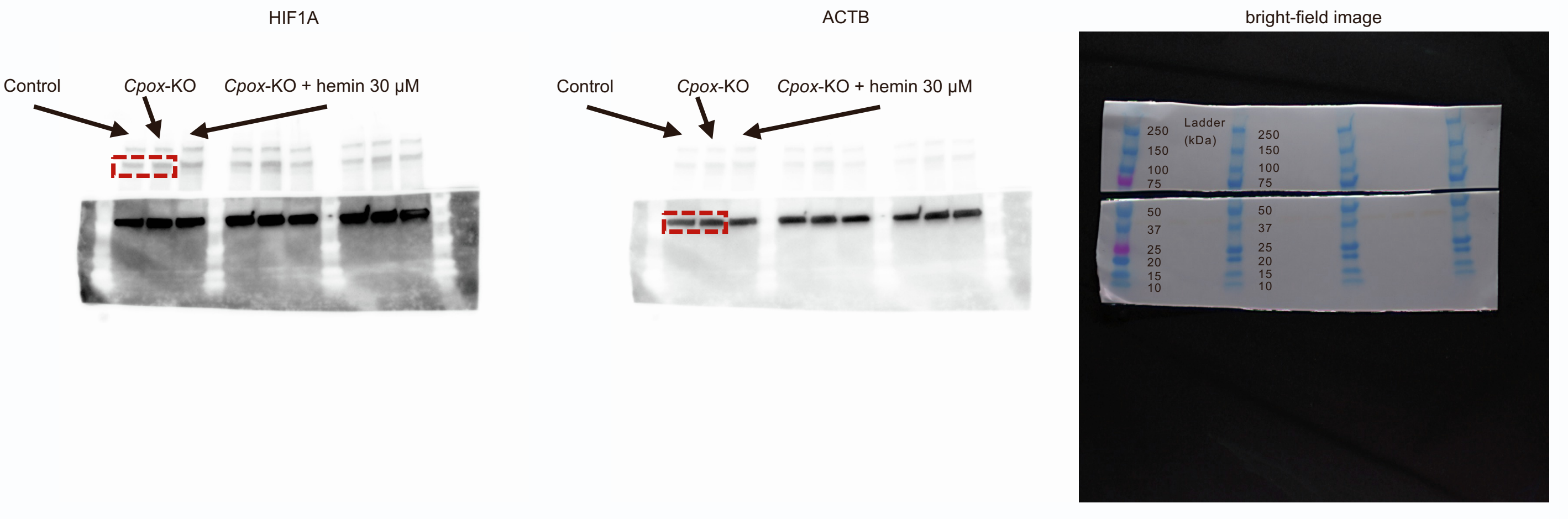
HIF1A
ACTB
bright-field image
Control
Cpox-KO
Cpox-KO + hemin 30 μM
Control
Cpox-KO
Cpox-KO + hemin 30 μM
Ladder
(kDa)
250
150
100
75
50
37
25
20
15
10
250
150
100
75
50
37
25
20
15
10

SUPPLEMENTAL DATA LEGENDS

Data S1. Uncropped original western blot images corresponding to Figure S5A and S6A.

(A) Uncropped original western blot images corresponding to Figure S5A. The chemiluminescence (left, middle) and the bright-field images (right) were obtained from the same membrane. The dashed boxes indicate the region corresponding to the OXPHOS complex component proteins (left) and ACTB (middle) band of control, *Cpox*-KO or *Cpox*-KO + hemin 30 μM used in Figure S5A.

(B) Uncropped original western blot images corresponding to Figure S6A. The chemiluminescence (left, middle) and the bright-field images (right) were obtained from the same membrane. The dashed boxes indicate the region corresponding to the HIF1A (left) and ACTB (middle) band of control or *Cpox*-KO used in Figure S6A.
