# Supplementary material for: Cell intrinsic heme synthesis is required for the proper activation of naive CD4+ T cells
Source: iScience. 2026 Nov 4;29(11):118114. doi: 10.1016/j.isci.2026.118114 (PMC13753341; doi:10.1016/j.isci.2026.118114)
Supplement: Supplementary file 2 [file mmc2.pdf]

Table S1. Cell Surface Markers defining each T cell subset

T cell subsets	Surface cell markers
DN3 cell	$CD4^-$, $CD8^-$, c-kit$^-$, $CD25^+$
DN4 cell	$CD4^-$, $CD8^-$, c-kit$^-$, $CD25^-$
Naive $CD4^+$ cell	$CD4^+$, $CD25^-$, $CD62L^{high}$, $CD44^{low}$
Effector Memory $CD4^+$ cell	$CD4^+$, $CD25^-$, $CD62L^{low}$, $CD44^{high}$
Regulatory $CD4^+$ cell	$CD4^+$, $CD25^+$

Table S2. Gene lists of common in three groups, related to Figure 6B and Figure S6D

(A)

Ahnak	*Hk2*	*Slc2a1*
Emp1	*Il2rb*	*Sult5a1*
F2rl2	*Lamc1*	*Tiparp*
Frmd4b	*Ppp2r3a*	*Tnfrsf8*
H2-Dma	*Slamf7*	

(B)

Adap1	*Hk2*	*Ppp2r3a*
Ahnak	*Id2*	*Slamf7*
Ccl3	*Ifng*	*Slc2a1*
Ccr5	*Ly6c2*	*Tiparp*
F2rl2	*Maff*	*Tnfrsf8*

Table S3. qPCR Primer List

qPCR primer	Forward	Reverse
Actb	CGTTGACATCCGTAAAGACCTC	AGCCACCGATCCACACAGA
Cpox	TGCCGACTTCACTGTGGAC	TGCTTCCTCAGAAAGATTCCCA
Fech	CCACCAAACCCCAAGCTCAA	GTTTGGCGATGAATGGTGCC
Hk2[40]	TGATCGCCTGCTTATTCACGG	AACCGCCTAGAAATCTCCAGA
Hmbs	GGCGGAGTCATGTCCGGTAA	AATCTTGTCTCCCGTGGTGG
Slc2a1	CAGCAGCTGTCGGGTATCAA	ATGGTCATGAGCACAGCACA
Urod	ATGGCTCTACCAAAGGCCAC	ACGAATGTAGGGCAGTGCAA

Supplemental Table Reference

40. Shen, H., Ojo, O.A., Ding, H., Mullen, L.J., Xing, C., Hossain, M.I., Yassin, A., Shi, V.Y., Lewis, Z., Podgorska, E., et al. (2024). HIF1α-regulated glycolysis promotes activation-induced cell death and IFN-γ induction in hypoxic T cells. Nat Commun *15*, 9394. 10.1038/s41467-024-53593-8.
